# Supplementary material for: Interplay between electrochemical reactions and mechanical responses in silicon–graphite anodes and its impact on degradation
Source: Nat Commun. 2021 May 11;12:2714. doi: 10.1038/s41467-021-22662-7 (PMC8113583; doi:10.1038/s41467-021-22662-7)
Supplement: Supplementary file 1 — Supplementary Information [file 41467_2021_22662_MOESM1_ESM.pdf]

Supplementary Information

Interplay between electrochemical reactions and mechanical responses in silicon–graphite anodes and its impact on degradation

Junhyuk Moon[1†]*, Heung Chan Lee[1†]*, Heechul Jung[1,5], Shinya Wakita[1,6], Sungnim Cho[1,6], Jaegu Yoon[1,6], Joowook Lee[1,6], Atsushi Ueda[1,7], Bokkyu Choi[1,6], Sihyung Lee[1], Kimihiko Ito[2], Yoshimi Kubo[2], Alan Christian Lim[3], Jeong Gil Seo[3], Jungho Yoo[4], Seungyeon Lee[1], Yongnam Ham[1], Woonjoong Baek[1], Young-Gyoon Ryu[1,6]* & In Taek Han[1]

[1]Samsung Advanced Institute of Technology, 130 Samsung-ro, Yeongtong-gu, Suwon-si, Gyeonggi-do 16678, Korea. [2]C4GR-GREEN, National Institute for Materials Science, 1-1 Namiki, Tsukuba, Ibaraki 305-0044, Japan. [3]Department of Chemical Engineering, Hanyang University, 222 Wangsimni-ro, Seongdong-gu, Seoul 04763, Korea. [4]National NanoFab Center, Daejeon 34141, Korea. [5]Present address: Department of Energy and Mineral Resources Engineering, Dong-A University, Bumin Campus, 225 Gudeok-ro, Seo-gu, Busan 49236, Korea. [6]Present address: Samsung SDI, 130 Samsung-ro, Yeongtong-gu, Suwon-si, Gyeonggi-do 16678, Korea. [7]Present address: Asahi Kasei Corporation, 2-1, Samejima, Fuji-shi, Shizuoka 416-8501, Japan.

[†]These authors contributed equally: Junhyuk Moon, Heung Chan Lee

*Correspondence to Junhyuk Moon (jh.d.moon@samsung.com), Heung Chan Lee (hchan.lee@samsung.com), and Young-Gyoon Ryu (ygryu@samsung.com).

Contents

Methods. Electrode preparation **(5)**

1. **Quantification of Li^+ content in individual materials** **(9)**
2. **Origin of abrupt changes in XRD signals** **(11)**
3. **Calculation of pressure on graphite** **(12)**
4. **Thermodynamic calculations for internal redox reaction** **(12)**
5. **Increase in CV charging time in cells using a silicon–graphite anode** **(15)**
6. **Change in thickness of cell and compression force on cell during CV charging** **(15)**
7. **Calculation of Li^+ penetration into silicon core during CV charging** **(16)**
8. **Determination of lithium contents** **(17)**
9. **Transmission electron microscopy (TEM)** **(19)**
10. **X-ray photoelectron spectroscopy (XPS)** **(21)**
11. **Electron probe micro-analyser results** **(22)**
12. **Effect of C-rate on anode lithiation** **(22)**
13. **Retardation of Li^+ transfer to silicon** **(23)**
14. **Image analysis** **(23)**
15. **DC–IR measurements** **(24)**
16. **Improvement over existing electrode design** **(24)**
17. **Utilisation of active materials in anode** **(26)**
18. **Direct visualisation through *in situ* electrochemical TEM** **(26)**

Supplementary Figures & Tables **(29)**

Supplementary References **(56)**

Supplementary Figure 1. Electrochemical properties of SSC composite. 29

Supplementary Figure 2. Effect of graphite hardness on cycle performance. 29

Supplementary Figure 3. Deconvolution of the mixed state of Cell B at 0.5C. 30

Supplementary Figure 4. Potential profiles of composite anode and individual components. 31

Supplementary Figure 5. Thermodynamic driving force corresponding to the XRD results during CV charging and resting of Cell B after operation at 0.5C. 32

Supplementary Figure 6. Amount of Li^+ in silicon and graphite during the first cycle at various C-rates. 33

Supplementary Figure 7. Cross-sectional SEM images of anodes during cycling. 34

Supplementary Figure 8. Amounts of lithium in anode determined by ICP-AES. 35

Supplementary Figure 9. Lithium contents in the cell, cathode, anode, and electrolyte. 36

Supplementary Figure 10. Lithium loss in cathode and anode *vs.* capacity retention. 37

Supplementary Figure 11. Formation of SEI on SSC in Cell B after discharge of first cycle. 38

Supplementary Figure 12. SEI on SSC and Li^+ accumulation in SSC of Cell B after discharge of 100th cycle. 39

Supplementary Figure 13. SEI chemical composition on cycled anodes of Cell B. 40

Supplementary Figure 14. EPMA characterisation of SEI in Cell B. 41

Supplementary Figure 15. SOCs in individual materials during charging at C-rates of 1C and 2C at the first cycle. 42

Supplementary Figure 16. SOCs in individual materials at the 250th cycle. 43

Supplementary Figure 17. Raw XRD data for Cell B. 44

Supplementary Figure 18. Evolution of pressure on graphite owing to volume expansion of Li_xSi in Cell B. 45

Supplementary Figure 19. Charge capacities of graphite and silicon at the first and 250th cycles. 46

Supplementary Figure 20. DC–IR characteristics of prismatic cells with a capacity of 8.7 Ah. 47

Supplementary Figure 21. Initial silicon oxide content measured by TEY- and PFY-XANES spectroscopy. 48

Supplementary Figure 22. Change in electrode design. 49

Supplementary Figure 23. Specimen for *in situ* electrochemical TEM. 50

Supplementary Figure 24. Potential waveform used to observe lithiation in the silicon–graphite anode and corresponding current. 51

Supplementary Figure 25. Electrochemical measurement during *in situ* electrochemical TEM. 52

Supplementary Figure 26. *In situ* electrochemical TEM characterisation of silicon–graphite during lithiation. 53

Supplementary Table 1. Lithium contents in the cathode, anode, and electrolyte of Cell B. 54

Supplementary Table 2. Information on cells. 55

Supplementary Methods. Electrode preparation

SSC. The surface-treated silicon/carbon (SSC) composite was prepared as follows. Silicon nanoparticles with lengths of 85 or 100 nm (denoted as 85- and 100-nm Si, respectively; see Table 1 for the average dimensions) were prepared by ball milling of micron-sized silicon powder. A chemical vapour deposition process was then used to deposit carbon on the surfaces of the silicon particles using a custom rotary kiln. An additional surface treatment was carried out with coal tar pitch as the carbon source, whereby the silicon particles and coal tar pitch were mixed in a powder mixer and heated at 900°C for 3 h to complete the carbonization reaction. This coated structure provides highly conductive percolation pathways and limits any unwanted reactions between the silicon particles and electrolyte.

The synthesised SSC particles measured approximately 5–15 µm, as observed by scanning electron microscopy (SEM; Helios NanoLab 450HP, FEI). The specific capacity of the SSC was 1,480 mAh g^{-1}, as determined using lithium half cells with the SSC as the working electrode at a delithiation rate of 0.2C. The areal capacity of the electrode was 1.5 mAh cm^{-2}. The anode materials in the half cells were tested under constant current–constant voltage (CC–CV) lithiation and CC delithiation modes between 1.5 and 0.01 V with a 0.05C cut-off at 25°C, following an initial formation cycle in the potential range of 10 mV to 1.5 V at 0.1C and full delithiation to 1.5 V. The measured capacity of the SSC was statistically the same as the theoretical capacity of the silicon embedded in the SSC. Hence, the capacity of the carbon and oxides in the SSC can be ignored. The content of silicon in the SSC was crosschecked by three different analysis methods: inductively coupled plasma atomic emission spectroscopy (ICP–AES; ICPS-

8100, Shimadzu), thermal gravimetric analysis (TGA; TGA/DSC2, Mettler Toledo), and oxygen–nitrogen elemental analysis (EMGA-920, Horiba).

Anode. Anodes with areal capacities of 4.4 and 6 mAh cm^{-2} were fabricated by roll-to-roll coating of an 8-μm-thick copper foil using a slurry comprising SSC particles (14.9 and 16.5 wt%, respectively), graphite (82.1 and 80.5 wt%, respectively), and binder (3 wt% each; modified lithium polyacrylate/polyvinyl alcohol, M_w = 500,000, Sumitomo) in deionised water. Graphite pellets with high (1.62 g mL^{-1}) and low (1.47 g mL^{-1}) densities were used, which were prepared by pressing two different graphite powders (denoted as HPD- and LPD-graphite, respectively) at 15.4 MPa for 30 s. HPD-graphite was prepared from a mixture of 80 wt% natural graphite (JFE Chemical Corp.) and 20 wt% synthetic graphite (Jiangxi Zichen Tech. Co.), while LPD-graphite was prepared from synthetic graphite (Shanshan Technology).

The difference in pellet density is related to the hardness of the graphite powder particles[1]. Typically, synthetic graphite is harder than natural graphite. To better understand the relationship between the type of graphite powder and pellet density, we measured the pressure-induced changes in volume of the HPD and LPD graphite powders, as well as two other graphite powders (denoted as graphite1 (Shanshan Technology) and graphite2 (JFE technology)), at different compaction pressures. The densities of the graphite pellets varied with the compaction pressure, as shown in Supplementary Fig. 2a. In addition, the graphite type greatly affected the compact density. The higher density of the HPD-graphite compact compared to the LPD-graphite compact indicates that LPD-

graphite is harder than HPD-graphite. The graphite1 and graphite2 compacts were both denser than the HPD- and LPD-graphite compacts.

To test the effect of graphite type on the cycling performance, anodes were prepared using graphite1, graphite2, HPD-graphite, and LPD-graphite (82.1 wt%) with 100-nm Si SSC (14.9 wt%) and binder (3 wt%; modified lithium polyacrylate/polyvinyl alcohol, M_w = 500,000, Sumitomo). The cycling performance of the prepared anode materials was tested using mini-18650 cells, which are essentially cylindrical-type 18650 cells with a hollowed-out cylindrical polypropylene (PP) insert. The mini-18650 cells comprised one double-side-coated cathode (dimensions of 54 × 200 mm) and one double-side-coated anode (dimensions of 57 × 240 mm) separated by a ceramic-coated separator film (Toray). The cathode powder comprised $Li_{1.0}Ni_{0.6}Co_{0.2}Mn_{0.2}O_2$ (80 wt%) and $Li_{1.0}Ni_{0.8}Co_{0.1}Al_{0.1}O_2$ (20 wt%). The cells were filled with 1.15 M $LiPF_6$ dissolved in fluoroethylene carbonate (FEC, Panax E-Tec), ethylene carbonate (EC, Panax E-Tec), ethylmethyl carbonate (EMC, Panax E-Tec), and dimethyl carbonate (DMC, Panax E-Tec) in a volume ratio of 7:7:46:40 (FEC/EC/EMC/DMC) as electrolyte. The areal capacity and n/p ratio of each of these cells were 3.4 mAh cm^{-2} and 1.03, respectively. The loading of the cathode and anode materials were 32.54 and 14.98 mg/cm^2, respectively, and the electrode densities were 3.6 and 1.7 g/cm^3, respectively. The formation and standard schemes were 0.1C and 0.2C CC–CV charging and CC discharging modes, respectively, between 4.2 and 2.8 V with a cut-off of 0.05C at 25°C. The mini-18650 cells were then cycled using 1C CC–CV charging and CC discharging modes between 4.2 and 2.8 V at 25°C. The cycling performance is shown in

Supplementary Fig. 2b, which indicates that lower pellet density (harder graphite) provides better cycling performance.

The anodes were dried under vacuum at 130°C for 3 h and roll-pressed at room temperature in a custom-made line. The specific capacities of the SSC–graphite working electrodes with areal capacities of 4.4 and 6 mAh cm^{-2} were 508 and 520 mAh g^{-1}, respectively; the corresponding electrode densities were 1.7 and 1.67 g cm^{-3}, respectively.

NCM. $Li_{1.0}Ni_{0.88}Co_{0.08}Mn_{0.04}O_2$ (NCM) powder was prepared by mixing co-precipitated $Ni_{0.88}Co_{0.08}Mn_{0.04}(OH)_2$ powder with $LiOH·H_2O$ and calcining the mixture at 750°C for 40 h in O_2 in a custom roller hearth kiln (RHK). The washing process involved stirring the prepared NCM powder in deionised water (weight ratio of 1:3) with a mechanical stirrer for 10 min, followed by filtration within 5 min. The NCM powder was placed in an air convection oven at 720°C overnight to evaporate any remaining water, followed by heat treatment at 720°C for 5 h under O_2 flow using the RHK. The specific capacity of the NCM was 214.8 mAh g^{-1}, as measured by half-cell discharge tests with CC–CV charging and CC discharging at 0.2C between 4.3 and 2.8 V with a 0.05C cut-off at 25°C.

Cathode. The cathode was fabricated by roll-to-roll coating of a 10-μm-thick aluminium foil using a slurry comprising NCM powder (97 wt%), carbon black (1.5 wt%, Cabot Co.), and polyvinylidene difluoride (PVDF; 1.5 wt%, Solef) in *N*-methyl-2-pyrrolidone (NMP, Sigma-Aldrich). The electrode was roll-pressed and dried under vacuum at 120°C in a custom-made line. The electrode density of the cathode was 3.6 g cm^{-3}.

Supplementary Note 1. Quantification of Li^+ content in individual materials

X-ray diffraction (XRD) spectra and electrochemical data were measured simultaneously. The diffraction spectra of graphite and the Cu current collector were extracted in the range of $1.45 < q < 2.0$ Å^{-1} and then fitted using OriginPro® to deconvolute the peaks of different graphite phases and determine the peak intensities. Here, q is the scattering wave vector. Previous crystallographic data for graphite[2–6] were referred to for the fitting.

The Coulombic efficiencies of the full cells were as high as 99.8%; thus, we ignored lithium loss due to solid electrolyte interphase (SEI) formation and electrode deterioration in a single cycle. Li^+ was therefore considered to be present only in the graphite and silicon components. We employed the following linear equation based on the assumption that the XRD intensity of each phase is linearly proportional to the Li^+ content of each phase.

$$\begin{pmatrix} A_i & \cdots & E_i \\ \vdots & \ddots & \vdots \\ A_f & \cdots & E_f \end{pmatrix} \begin{pmatrix} \alpha \\ \vdots \\ \varepsilon \end{pmatrix} + \begin{pmatrix} \varphi_i \\ \vdots \\ \varphi_f \end{pmatrix} = \begin{pmatrix} Capacity_i \\ \vdots \\ Capacity_f \end{pmatrix}, \qquad (1)$$

where A_k, B_k, C_k, D_k, and E_k are the intensities of the peaks for stage 1, stage 2L, stage 2, stage 3L, and stage 4L, respectively, which are extracted from the fitting results of the XRD analysis; α, β, γ, δ, and ε are the corresponding coefficients that convert the intensity of each phase into its capacity. φ_k is the Li^+ content in silicon, and $Capacity_k$ is the capacity of a cell in the kth sequential data. The stages are named by the order of lithium ions in each nth interslab of graphite, and the letter L indicates 'liquid-like', without any in-plane order[7]. The lithium contents in stage 2L and stage 2 are summed and noted as stage 2 in the main text.

The amount of Li^+ in silicon can be determined by considering Supplementary Equation (1). First, the coefficients α, β, γ, δ, and ε were extracted from the *operando* XRD results of a full cell with a graphite-only anode and high-nickel cathode, denoted as Cell A, by exploiting the pseudo-inversion method[8] (Supplementary Fig. 3a). No assumptions were required for this step. Second, the XRD profiles were normalised using the intensity of the Cu(111) peak. Subsequently, we calculated the amount of Li^+ in graphite in a full cell with a silicon–graphite anode, denoted as Cell B (Supplementary Fig. 3b). Finally, the amount of Li^+ in silicon in Cell B was obtained (Supplementary Fig. 3c). Our only ansatz here is that the XRD intensity is proportional to the capacity of the graphite phase. The baselines were generated by the spline method and Gaussian multi-peak fitting was conducted using a nonlinear curve fitting tool. The number of points for baseline generation was the only manually selected data and was approached with great caution within the series analysis.

This method was used to devise the amounts of Li^+ in each component, as shown in Fig. 1a. The weight of each material in the anode was determined based on the weight ratios of SSC and graphite in the anode and the content of silicon in the SSC, which are described in Supplementary Methods 1. The loading of the anode was 17.84 mg cm^{-2}, and the silicon/carbon/oxygen weight ratio was 48.3/47.7/4 (*i.e.* the weight ratio in SSC). When silicon is fully lithiated, the phase of lithium silicide is $Li_{3.75}Si$. Thus, the weights of silicon and graphite in Cell B are 0.07457 and 0.85113 g, respectively. Therefore, the theoretical capacities for fully lithiated silicon and graphite in Cell B are 266.97 (= 3580 mAh g^{-1} × 0.07457 g) and 318.32 mAh, respectively.

The initial Coulombic efficiencies of SSC and graphite are 86.4% and 93%, respectively. The state-of-charge (SOC) of each material can be calculated using these reversible capacities. At point c in Fig. 1a, the capacities in silicon and graphite are 184.53 and 195.59 mAh, respectively. The SOCs of the silicon, graphite, anode, and cell are 79.98%, 66.07%, 72.16%, and 88.35%, respectively. At point d, the capacities in silicon and graphite are 151.45 and 278.81 mAh, respectively, and the SOCs of the silicon, graphite, anode, and cell are 65.64%, 94.18%, 81.68%, and 100%, respectively.

Supplementary Note 2. Origin of abrupt changes in XRD signals

The differential capacity (dQ/dV) plots of Li–Si alloys typically exhibit much broader peaks than those of graphite, which indicates that various alloy phases exist simultaneously in the silicon component. The silicon component experiences an increase in pressure due to volume expansion upon lithiation, which hinders the insertion of Li^+ into the silicon during charging. This may promote the formation of a gradient of phases in the Li–Si alloy. Because we used small silicon particles with a short-axis length of approximately 30 nm, the gradient of phases in the Li–Si alloy is expected to be small in a cycle.

The amount of Li^+, n, *vs.* the potential of silicon, E, near the phase-transition point, E^*, has the form $-u(x)$, where $u(x)$ is a step function, so that the asymptotic form of n *vs.* E is $\tan^{-1}x$. The average amount of Li^+ in the Li–Si alloy, $\langle n \rangle$, can be described as

$$\langle n \rangle = -\frac{\partial G}{\partial \mu} \propto -\frac{\partial\big(n(E-E^*)\big)}{\partial E}\bigg|_{E \approx E^*} \sim \frac{\mathrm{d}\tan^{-1}(E-E^*)}{\mathrm{d}E}\bigg|_{E \approx E^*} = \frac{1}{(1+(E-E^*)^2)}\bigg|_{E \approx E^*}. \quad (2)$$

Sudden changes in the individual SOCs were clearly observed in our XRD results for the following reasons. When a silicon phase transition occurs, the amount of Li^+ in the

Li–Si alloy changes abruptly. According to this argument, the change in the amount of Li^+ is more abrupt than the change in potential that occurs near the phase transition. Furthermore, the amount of Li^+ is directly associated with the diffraction peaks measured by XRD.

Supplementary Note 3. Calculation of pressure on graphite

When lithiation in stage 1 graphite is relatively small, the fitting error of the peak's position is larger because the signal is small. From the XRD peak shift, the pressure applied on the graphite (ΔP) can be calculated using the following equation:

$$\Delta P \approx \frac{1}{\kappa_c}\frac{c_0 - c}{c_0}, \tag{3}$$

where κ_c is the compressibility of the c-axis of LiC_6, reported to be $1/c_o(\partial c/\partial P)_{P_o} = 1.344 \times 10^{-2}$ GPa^{-1}; c is the c-axis constant; and c_0 is the c-axis lattice constant at the reference pressure, P_0 [(9)].

Supplementary Note 4. Thermodynamic calculations for internal redox reaction

The Li_xC_6 phase can be determined from the amount of Li^+ in graphite, while the phase of the silicon particle shell can be identified using the position of the phase transition from the XRD results. It is difficult for Li^+ in the shell of the silicon particles to move into the core because of an increase in pressure due to volume expansion of the lithiated shell[10]. Thus, the chemical potential of the silicon component reduces more slowly than that of the graphite[11]. For this reason, we assume in this work that graphite interacts only with the shell of the silicon particles. The difference in chemical potentials ($\Delta\mu$) at a constant temperature and pressure can therefore be described by the following equation:

$$\Delta\mu = \left(\mu_{\mathrm{Li}_y\mathrm{C}_6} - \mu_{\mathrm{Li}}\right) - \left(\mu_{\mathrm{Li}_x\mathrm{Si}_{\mathrm{shell}}} - \mu_{\mathrm{Li}}\right) = \left(\frac{\partial G_{\mathrm{Li}_y\mathrm{C}_6}}{\partial \mathrm{y}}\bigg|_{T,P} - G_{\mathrm{Li}}\right) - \left(\frac{\partial G_{\mathrm{Li}_x\mathrm{Si}_{\mathrm{shell}}}}{\partial x}\bigg|_{T,P} - G_{\mathrm{Li}}\right), \quad (4)$$

where μ_i and G_i are the chemical potentials and Gibbs free energies of the components, respectively (i = Li_xSi_{shell}, Li_yC_6, and metallic Li)[12, 13]. Herein, Li_xSi_{shell} is the lithium silicide phase of the shell of the silicon particles.

The chemical potential is the change in Gibbs free energy that occurs as the amount of Li^+ changes. In the case of charging Cell B at 0.5C (see Fig. 1b), the third phase transition (red triangle at 65% SOC) corresponds to the phase transition from $Li_{2.3}Si$ to $Li_{3.25}Si$[14, 15], which occurs mostly on the surface of silicon due to the two-phase lithiation. The ratio of the electrochemically active volume (presumably the surface) to the total volume of the silicon particle can be calculated as 92.3% from the difference between the Li^+ content in the surface phase ($Li_{3.25}Si$) and the measured total Li^+ content in silicon during the third phase transition. Assuming that the reacted volume is maintained after the third phase transition, the lithiated state of the silicon particle shell can be estimated, and subsequently, the chemical potentials of the Li_xSi_{shell} and Li_yC_6 phases can be calculated using previously reported thermodynamic values[15–17]. Here, the pressure and temperature of this system were set to 1 atm and 297 K, respectively. In order to correct for the pressure effect, we assume that the changes in the internal energy and entropy owing to the volume change can be ignored. Therefore, the change in the chemical potential can be described by the following equation:

$$\Delta\left(\frac{\partial G}{\partial x}\right)_{P,T} = P\left(\frac{\partial V}{\partial x}\right)_{P,T} - P_0\left(\frac{\partial V}{\partial x}\right)_{P_0,T} \approx (P - P_0)\left(\frac{\partial V}{\partial x}\right)_{P_0,T}, \quad (5)$$

where P, V, and x are pressure, volume, and number of lithium ions, respectively.

In graphite, the change in the c-axis dimension owing to applied pressure is smaller than the difference between the c-axes of LiC_6 and graphite; thus, the maximum of

$(\partial V/\partial x)_{P,T}$ can be calculated. If the increase in pressure is 0.4 Gpa, the difference in the chemical potential of Li_yC_6 owing to the increase in pressure can be calculated as follows:

$$\Delta\left(\frac{\partial G_{\mathrm{Li}_y\mathrm{C}_6}}{\partial y}\right) \leq (4 \times 10^8\ \mathrm{Pa}) \times (3.25 \times 10^{-30}\ \mathrm{m}^3) \times N_\mathrm{A} = 0.728\ \mathrm{kJ/mol}, \qquad (6)$$

where N_A is Avogadro's constant.

In the same manner, the maximum of $(\partial V/\partial x)_{P,T}$ can be described as $(\partial V/\partial x)_{P,T}$ = (6.662 + 1.261x) cm^3 mol^{-1}, where x corresponds to x in Li_xSi using the volume change of silicon as a function of the SOC[18]. Thus, the difference in the chemical potential of the Li_xSi_{shell} phase owing to the increase in pressure at x = 3.0 can be described as

$$\Delta\left(\frac{\partial G_{\mathrm{Li}_x\mathrm{Si}_{\mathrm{shell}}}}{\partial x}\right) \leq (4 \times 10^8\ \mathrm{Pa}) \times (1.045 \times 10^{-5}\ \mathrm{m}^3/\mathrm{mol}) = 4.18\ \mathrm{kJ/mol}. \qquad (7)$$

Therefore, upon an increase in pressure of 0.4 Gpa, the chemical potentials of graphite and silicon would decrease by −7.545 and −43.33 mV, respectively. Supplementary Fig. 5 summarises these processes for calculating the thermodynamic driving force during a CV charge and rest cycle. In this calculation, the increase in pressure was calculated using the XRD peak shift at stage 1. The upper-most panel of Supplementary Fig. 5 shows the driving force of the internal redox couple. Li^+ ions can move from silicon to graphite during CV charging and resting. All potential difference ($\Delta\mu$) values (blue circles) in the upper-most panel in Supplementary Fig. 5 are below 0 kJ mol^{-1}. Our situation differs from the conventional solid-solution model for homogenous states; but is well described by Bazant's report on non-equilibrium and inhomogeneous states[19].

In the half-cell experiments, no internal redox couples were observed. Lithiation in the working electrode is regulated by a constant current until a set potential (0.01 V), at which point the lithium content in Li_xSi reaches x = 3.75[(20)]. Lithiation in the anode of the

full cell, however, is regulated by the overall potential difference between the cathode and anode. The minimum potential of the anode (~0.1 V) during full-cell charging, as measured using a three-electrode set-up, was higher than that measured in the half-cell experiment, as shown in Fig. 2a. Therefore, the electric potential applied to the anode in the full cell during charging was higher than that in the half cell.

Supplementary Note 5. Increase in CV charging time in cells using a silicon–graphite anode

The internal redox reaction in the silicon–graphite anode leads to an increase in the CV charging time. After CC charging is terminated, the highly concentrated lithium at the anode–electrolyte interface diffuses into the bulk of the anode. Moreover, CV charging is terminated when the concentration gradient within the active material is minimised[21]. There is a distinctive pathway for minimizing the concentration gradient, namely Li^+ mass transport from silicon to graphite *via* the internal redox reaction. During CV charging, this pathway delays the end of charging because Li^+ diffusivity in silicon is two orders less than that in graphite[22, 23]. Consequently, the time for CV charging increases, and the delithiated cathode experiences a high cut-off voltage during the extended CV charging time.

Supplementary Note 6. Change in thickness of cell and compression force on cell during CV charging

As shown in Fig. 1, Li^+ transfers from silicon to graphite during CV charging. Thus, the thickness of the anode, which increases during CC charging, must decrease from a certain

point during CV charging owing to the large variation in the size of (de)lithiated silicon. We monitored the variation in thickness over a cycle by measuring the thickness *in situ* (see Methods in the main text for details). During the CV charging and rest stages, which correspond to the voltage plateaus in Fig. 1d, the thickness of the cell using the silicon–graphite anode (upper panel of Fig. 1d) starts to reduce after 251 min, whereas the thickness of the graphite-only anode (lower panel of Fig. 1d) plateaus after 255 min.

Simultaneously, the force on the cell with the silicon–graphite anode (upper panel of Fig. 1e) steeply increases until 127 min, and then continuously decreases during the CV charging and rest stages. In contrast, the force on the cell with the graphite-only anode (lower panel of Fig. 1e) reduces only slightly during the rest stage. This result agrees well with the thickness change during CV charging.

The thickness and force variation during CV charging do not originate from particle rearrangement, as this would be illustrated in the cross-sectional images at 0% and 100% SOC every 50 cycles shown in Supplementary Fig. 7. Supplementary Fig. 7 demonstrates that graphite is in a compacted state; that is, the space between the SSC and graphite remains unfilled in the 0% SOC images.

Supplementary Note 7. Calculation of Li^+ penetration into silicon core during CV charging

Here, free energy of Li^+ penetrating into the silicon core during CV charging is calculated by using an analytical solution based on two-phase lithiation, which describes the effect of stress on the driving force for the reaction[24]. At the interface between the Li_xSi shell and core of a spherical silicon particle, the net change in free energy is described as

$$\Delta G = \Delta G_\mathrm{r} - e\phi + \frac{1}{x}\left(\sigma_m^{\mathrm{Si}} V_{\mathrm{Si}} - \sigma_m^{\mathrm{Li}_x\mathrm{Si}} V_{\mathrm{Li}_x\mathrm{Si}}\right) = \Delta G_\mathrm{r} - e\phi + \frac{2\sigma_\mathrm{Y} V_{\mathrm{Si}}}{x}\left[(\beta - 1)\ln\left(\frac{b}{a}\right) + \frac{\beta}{3}\right], \quad (8)$$

where ΔG_r is the free energy of the reaction in which one lithium atom and $1/x$ silicon atoms react and form Li_xSi when both the stress and the applied voltage vanish; e is the elementary charge; ϕ is the voltage; σ_m^{Si} and $\sigma_m^{\mathrm{Li}_x\mathrm{Si}}$ are the mean stress in silicon and Li_xSi at the interface, respectively; V_{Si} and $V_{\mathrm{Li}_x\mathrm{Si}}$ are the volumes per unit of Si and Li_xSi, respectively; β is the ratio of the volume of the lithiated shell over the shell's volume before lithiation; σ_Y is the yield strength of the lithiated phase; and a and b are the radii of the lithiated particle and the particle core.

For this calculation, we adopted the following values. (i) Before CV: $\Delta G_r = -24.3$ kJ mol^{-1}, $e\phi = 11.578$ kJ mol^{-1}, $\beta = 3.325$, $x = 3.2$, $\sigma_Y = 1$ GPa[25], and $V_{Si} = 11.944$ cm^3 mol^{-1}; (ii) during CV: $\Delta G_r = -30.79$ kJ mol^{-1}, $e\phi = 9.65$ kJ mol^{-1}, $\beta = 2.807$, and $x = 2.6$. ΔG_r was calculated using previously reported thermodynamic values[15–17]; $e\phi$ was obtained from the three-electrode data in Fig. 2a; and β and V_{Si} were calculated using previously reported values[18].

Before CV charging, the value of a/b is 0.2047 when ΔG is zero. During CV charging, ΔG becomes negative when a/b equals 0.2047, which indicates that Li^+ can penetrate into the depressurized silicon core during CV charging owing to Li^+ crosstalk.

Supplementary Note 8. Determination of lithium contents

The amounts of lithium in the electrodes and electrolyte were traced every 50 cycles by the following process. After discharging, the cells were disassembled in an Ar-filled glovebox. All electrodes were rinsed with DMC for 5 min and dried under vacuum for 6 h, and the remaining electrolyte was collected. After chemical treatment[26], the amounts of

lithium in the electrodes and electrolyte were determined using ICP–AES. Their weights were calculated using the loading level (g cm^{-2}) and area of the electrodes, and the amounts of lithium were converted into capacities. The weight of lithium in the electrolyte was calculated using the weight of electrolyte in the cell.

To measure the amount of electrochemically inactive lithium, a half cell assembled using the disassembled anode was fully delithiated to a cut-off voltage of 1.5 V at 0.05C. Then, the half cell was disassembled in an Ar-filled glovebox, and the amount of lithium in the anode (*i.e.* the electrochemically inactive lithium) was determined using ICP–AES. The difference in the amounts of lithium between the discharged anode and the reassembled-and-delithiated anode corresponds to the amount of Li^+ remaining in the anode. After the first and 250th cycles of Cell B, we measured the amounts of total lithium (black), inactive lithium (red), and remaining Li^+ (blue) in the anode, as shown in Supplementary Fig. 8. Here, inactive lithium corresponds to the lithium consumed during the formation of the SEI. The loading (g cm^{-2}) of the anode; the molar masses of carbon, silicon, and lithium; and the atomic composition of the anode were used to calculate the SOCs of anode after the first and 250th cycles, which were 3.5% and 14.3%, respectively.

Supplementary Table 1 describes these data sets. Supplementary Fig. 9 shows the lithium contents in the cell, cathode, anode, and electrolyte. The amounts of lithium in the cell (black open), anode (black filled), and electrolyte (green) of Cell B were measured every 50 cycles. The red open circles, red filled circles, and blue circles indicate the amounts of active lithium in the cathode, inactive lithium in the anode, and remaining Li^+ in the anode, respectively. The measured amount of total lithium in the cell is almost unchanged, which shows the reliability in measuring lithium using ICP–AES.

During cycling, the amount of lithium in the electrolyte decreased from 9.26 mg at the first cycle to 7.87 mg at the 250th cycle, while the inactive lithium content in the anode increases from 19.39 mg at the first cycle to 21.05 mg at the 250th cycle. The change in the lithium content in the electrolyte is equivalent to 84.2% of the change in inactive lithium content in the anode. Given the consumption of organic solvents, the lithium consumption due to SEI formation is associated with the loss of lithium from the electrolyte. Hence, the loss of lithium ions to form the SEI occurs mostly during the formation stage.

The remaining Li^+ in silicon increases 9.1-fold relative to the increase in inactive lithium during the cycling of Cell B. Because of this residual Li^+, increasing the inner volume of the silicon particles results in the formation of lithium silicate on the surface of the silicon, which leads to high resistance, as shown in Fig. 3b.

Supplementary Fig. 10 summarises the lithium loss in each component of Cell B during cycling. The active lithium content in the cathode (blue open circles) during full-cell cycling was obtained by subtracting the lithium content in the cathode at 0% SOC from that at 100% SOC. The amounts of total lithium (black filled circles), inactive lithium (red), and remaining Li^+ (blue) in the anode are shown.

Supplementary Note 9. Transmission electron microscopy (TEM)

Ex situ transmission electron microscopy (TEM) analyses were carried out using a JEM-ARM200F-G microscope (JEOL) with electron energy loss spectroscopy (EELS; Gatan Enfinium) and energy dispersive spectroscopy (EDS; SDD type) detectors. The cell was disassembled in an Ar-purged glovebox, and the electrode was rinsed in DMC for 5 min

and then dried under vacuum for 30 min. To avoid sample contamination and reaction upon air exposure, a vacuum transfer TEM holder (Gatan Model 648) and custom transfer vessel for FIB were used. All samples were moved from the FIB transfer vessel to the vacuum transfer TEM holder in an Ar-purged glovebox.

In scanning transmission electron microscopy (STEM) mode, the specimen is illuminated only at the position where the focused electron beam is transmitted, so EELS and EDS signals generated from electron excitation have high spatial accuracy. We investigated the chemical distribution of the SSC and SEI on SSC after the first and 100th cycles. The SEI, consisting mainly of LiF, grows during the formation stage on the coal tar pitch surrounding the SSC particles (see Supplementary Methods 1), as shown in Supplementary Fig. 11[27]. After 100 cycles, the Li-K edge EELS image exhibits bright and dark red areas, as shown in Supplementary Fig. 12b. The bright part surrounds the pitch, and consists mainly of LiF (Supplementary Fig. 12d), similarly to that on the SSC particles after the first cycle (Supplementary Fig. 11e). The dark part is lithium silicide (Supplementary Fig. 12d). Therefore, Li^+ had accumulated inside the silicon particles by the 100th cycle. These results agree well with the results in Supplementary Note 8, which illustrates the accumulation of Li^+ in silicon during long-term cycling.

Along with PFY-XANES result (Figs. 3c and f), this STEM-EELS results exhibit that LiF as a component of SEI forms mainly on the carbon components of SSC. The ratio of graphite and the carbon in SSC is 82.1 : 7.1 (= 14.9 wt% (wt% of SSC in anode) × 47.7% (carbon ratio in SSC)). During Cell B (capacity of 430 mAh) operation, the lithium losses by SEI at first and 250th cycles are 74.9 and 81.31 mAh, respectively (Supplementary Table 1). Then, the roughly estimated lithium losses by SEI on SSC at

first and 250th cycles are 6.0 and 6.5 mAh, respectively. The specific properties of SEIs on different components as well as the ratios of volumes and surfaces of materials could affect the calculation result, but might not significantly change the estimation.

Supplementary Note 10. X-ray photoelectron spectroscopy (XPS)

For *ex situ* XPS measurements (PHI Quantera-II), the core-level spectra were measured using Al Kα as the excitation source (1486.6 eV) at an accelerating voltage of 1 kV. All spectra were referenced to the *C 1s* peak at 284.8 eV. The cells were dissembled in an Ar-filled glovebox and rinsed in DMC for 5 min, followed by 30 min drying under vacuum. Subsequently, the electrodes were loaded into an in-house airtight vessel and transferred to the instrument without exposure to ambient air.

The chemical composition of the SEI in Cell B was investigated using XPS. The results are shown in Supplementary Fig. 13. The SEI surface consists of organic compounds such as ROLi (where R is a hydrogen, hydrocarbon side chain, or group of atoms) and inorganic compounds such as LiF, Li_2CO_3, and Li_2O. Supplementary Fig. 13 also exhibits a tendency for the LiF (684.8 eV), Li_2CO_3 (531.8 eV), and C–O–C (286.4 eV) components to increase with cycling. In addition, all the *C 1s* XPS spectra (Supplementary Fig. 13c) exhibit a peak at ~284 eV, which corresponds to the underlying graphite component of the anodes (the penetration depth of XPS is several nanometres). This suggests that the SEI layer is still thinner than the maximum XPS penetration depth of *ca.* 10 nm [(28)] after 250 cycles. Thus, the growth of the SEI was well controlled, which is in good agreement with the ICP results.

The formation of LiF is attributed to the decomposition of FEC (a component of the electrolyte). LiF forms on the outside of the SSC particles, where the coal tar pitch is situated. However, LiF is not observed inside the SSC particles, as shown in the TEM results in Supplementary Note 9. Thus, the SSC structure effectively hinders the electrolyte from penetrating into the silicon particles.

Supplementary Note 11. Electron probe micro-analysis (EPMA)

The SEI was visualised by measuring the distribution of P originating from the electrolyte across the cross-sectioned anodes using electron probe micro-analysis (EPMA; JXA 8530F, JEOL). The results are shown in Supplementary Fig. 14. The SEI formed on the outside of the active materials, graphite, and SSC during cycling. This result corroborates the ICP results.

Unlike previous studies on the breakage of graphite, whereby excessive volume expansion of the silicon phase in irreversible reactions with the electrolyte pressurise the graphite phase and cause complete graphite breakage[29], these ICP and EPMA results show that the breakage of graphite is limited. The structure of the SSC effectively prevents side reactions between the silicon component and electrolyte, as shown in Figs. 3b, c, e, and f.

Supplementary Note 12. Effect of C-rate on anode lithiation

The internal redox reaction also occurs at higher C-rates of 1C and 2C, as shown in Supplementary Fig. 15. At a low C-rate of 0.5C (Fig. 1b), the experimental individual SOCs are in good agreement with the calculated values. When the C-rate increases to 1C

and 2C (Supplementary Fig. 8a and b), the sudden changes in the amount of Li^+ due to Li_yC_6 phase transitions become less distinct, and the silicon SOC decreases, while those of graphite become higher at SOCs of below 67%. This behaviour might be because silicon has a poorer C-rate capability than graphite[30].

Supplementary Note 13. Retardation of Li^+ transfer to silicon

The calculated silicon SOCs (red line, Supplementary Fig. 16) indicate the intrinsic lithiation behaviour of the silicon component. The experimentally determined silicon SOCs for the 250th cycle (red circles, Supplementary Fig. 16) are in good agreement with the calculated values between 0% and 15%. However, at SOCs of 15%–34%, the experimental data deviate discontinuously from the calculated SOCs, and do not follow the intrinsic lithiation behaviour of silicon.

Supplementary Note 14. Image analysis

The features corresponding to the silicon boundaries were extracted from the grey-scale SEM micrographs using image analysis. First, the grey-scale SEM images were converted to binary images, and the coordinates associated with the boundaries were estimated. To preserve the silicon boundaries and edges where there were large changes in intensity while selectively removing uncertain lines with small intensity fluctuations, L_0 gradient minimisation[29] (with a weighting factor of 0.009) was used, which is particularly effective for highlighting silicon boundaries. Furthermore, morphological processing[32] (dilation/erosion) was used to preserve the original object shapes. Otsu's method[33] was used to segment silicon (white, '1') and others (black, '0'). When silicon

regions overlapped in the image, the silicon particles were partially manually divided. Finally, the MATLAB® 2018a built-in function 'regionprops' was applied to calculate the average particle area and diameter. All algorithms were implemented using MATLAB® 2018a (MathWorks, Inc.).

Supplementary Note 15. DC–IR measurements

The direct current–internal resistance (DC–IR) characteristics of the prismatic cells at 50% SOC were measured every 100 cycles. DC–IR measurements were conducted by applying a 10 s long negative current pulse of 1C to the cell after 1 h of rest at open-circuit voltage (OCV), and the resistance was calculated from the difference between the OCV and the potential at the end of the pulse, divided by the pulse current[34, 35]. The increase in the DC–IR values for cells with HPD-graphite was larger than that for cells with LPD-graphite, as shown in Supplementary Fig. 20.

Supplementary Note 16. Improvement over existing electrode design

Thus far, the anode has been designed based on the assumption that the Li_xSi phase would be x = 3.75 at full lithiation. In conventional electrode design, the $Li_{3.75}Si$ phase is difficult to attain in full-cell operation conditions, as the internal redox reaction dominates the anode reaction during CV charging, as shown in Fig. 1. Li^+ crosstalk facilitates long-term degradation of the cell. However, we can directly relieve the long-term detrimental effects of Li^+ crosstalk by changing the design of the anode, maintaining a fixed graphite content, and increasing the SSC content to 111% relative to that in Cell D. Because Li–Si alloys have a convex Gibbs free energy landscape as a function of x in

Li_xSi with a minimum at $x = 2.33$[15, 16], prevention of over-lithiation of silicon ($x < 2.33$) would directly reduce Li^+ crosstalk.

Based on the above consideration, a revised anode was prepared from 18 wt% SSC, 79 wt% LPD-graphite, and 3 wt% binder. With the revised design, which simply increased the SSC content to 111% compared to that in Cell D, the cross-points of the SSC and graphite SOCs increased from 72.9% to 78.8% anode SOC, as shown in Supplementary Fig. 22a. In addition, by changing the electrode design, the utilisation of active materials in the anode decreased from 87.3% to 82.8% (see detailed calculations in Supplementary Note 17). This change prevents over-lithiation of silicon, which is the origin of Li^+ crosstalk between silicon and graphite during CV charging.

Increasing the number of accommodation sites for lithium in silicon and the prevention of over-lithiation reduced Li^+ crosstalk during CV charging in the full cell. Consequently, the ratio of the time for CV charging to the total charging time (CV ratio) was reduced, and the capacity retention enhanced, as shown in Supplementary Fig. 22b. Furthermore, Supplementary Figs. 22c and d show that the potentials of the anode and cathode at the end of charging were almost unchanged with cycling; hence, the potential of the cathode does not increase upon increasing the silicon content.

With our new understanding of the Li^+ crosstalk degradation mechanism, we were able to enhance the cycling performance while minimizing adverse effects. The anode thicknesses in the previous and revised designs were 73.3 and 76.8 μm, respectively. Thus, the revised electrode design increases the electrode thickness by 5%. However, the change in thickness at full volume expansion decreases by 2.5% (from 123% to 120.5% *vs.* initial thickness). Simultaneously, the discharge capacity for the first cycle after the

formation stage decreases by 0.9% (from 606 to 600.5 mAh), but the capacity retention ratio increases from 95.4% to 97.1% after 100 cycles.

Supplementary Note 17. Utilisation of active materials in anode

The discharge capacity of the full cell, Q_c, is given as follows:

$$Q_\mathrm{c} = Q_0^+ - Q_\mathrm{I}^- = Q_0^+ - \frac{n}{p} Q_0^+ (1 - \varphi^-), \tag{9}$$

where Q_0^+, Q_I^-, n/p, and φ^- are the initial cathode charge capacity, the irreversible capacity of the anode, the negative to positive capacity ratio, and the initial Coulombic efficiency of the anode, respectively. Because the cathode has a lower cut-off voltage, the utilisation of the anode, β, can be expressed using the utilisation of the cathode, α:

$$\beta = \frac{\alpha Q_\mathrm{c}}{Q_\mathrm{R}^-} = \frac{\alpha Q_\mathrm{c}}{\frac{n}{p} Q_0^+ \varphi^-}, \tag{10}$$

where Q_R^- is the reversible capacity of the anode[36].

By changing the design of the electrode, the *n*/*p* ratio increases from 1.03 to 1.08, and the initial Coulombic efficiency (ICE) of the anode decreases from 88.9% to 88.7%. After the initial formation stage, the discharge capacity of the full cell decreases from 88.57% to 87.8% of the initial cathode charge capacity. Given the lower cut-off potential of the cathode (3.35 V), the utilisation of the cathode is 90.3%. Therefore, the utilisation of the anode decreases from 87.3% to 82.8%.

Supplementary Note 18. Direct visualisation through *in situ* electrochemical TEM

Recent developments in *in situ* TEM have provided a new opportunity to directly observe Li^+ diffusion between carbon (or graphite) and silicon[37–39]. However, this open-cell *in situ* TEM platform cannot reproduce the complete battery cell environment. A platform

with a sealed liquid cell that can mimic the authentic battery cell environment was employed in this study[40–45]. A cross-sectional silicon–graphite anode was fabricated (see the Experimental Set-up below), as shown in Supplementary Fig. 23. Bright-field TEM images were acquired during holds in the potential sweep. The potential waveform used in this observation and its corresponding current profile are shown in Supplementary Fig. 24. Linear sweep voltammograms from −1 to −3.94 V and a chronoamperogram at a constant potential of −3.94 V for lithiation in the silicon–graphite anode are shown in Supplementary Fig. 25. Supplementary Fig. 26 shows six sequential TEM images acquired at points a–f in Supplementary Fig. 24. The morphology of the silicon–graphite anode changes suddenly between Supplementary Figs. 26d and 26e, with a reduction in contrast between the silicon and graphite components, because Li^+ inserts into both the silicon and graphite. Supplementary Fig. 25d also clearly shows the corresponding electrochemical signals of lithiation in both silicon and graphite below −3.75 V, which demonstrates the Li^+ transport from graphite and/or carbon to silicon. In the next image, Supplementary Fig. 26f, the micrograph is hazy owing to SEI formation. The focused ion beam (FIB) fabrication exposes a new interface with the electrolyte, and the SEI forms on this interface. The potential of −3.94 V *vs.* Pt corresponds to below 0.1 V *vs.* Li/Li^+, whereby the SEI easily forms. Unfortunately, the potential at which Li^+ crosstalk occurs between silicon and graphite is similar to that for forming the SEI.

Experimental set-up. Two silicon microchips were sandwiched together in the tip of the *in situ* TEM holder (Poseidon Select, Protochips). The upper microchip (ECT-45CR-10, Protochips) contained a glassy carbon working electrode (WE) located over a silicon

nitride window with dimensions of 550 × 40 μm and thickness of 50 nm, as well as a circular Pt counter electrode (CE). This upper microchip has a spacer area of 500-nm-thick SU-8, which makes a channel through which the electrolyte is delivered to the electrochemical cell. The lower microchip (EPB-55A-10, Protochips) also has a silicon nitride window. With the cell fully assembled and sealed, TEM images of electrochemical reactions that occur on the WE can be recorded through the silicon nitride windows.

The cross-sectional specimen for TEM analysis was fabricated using a FIB (Helios NanoLab 600, FEI), and the prepared specimen was then placed onto the glassy carbon WE in the upper microchip through an *ex situ* lift-out technique[46]. Subsequently, the specimen was fixed using Pt deposition welding. The *in situ* TEM holder was connected to a potentiostat (Reference 600+, Gamry), which enabled elaborate electrochemical analysis. The electrolyte, 1.15 M $LiPF_6$ dissolved in EC, EMC, and DMC in a volume ratio of 2:4:4, was introduced through the holder and between the microchips with microfluidic polyether ether ketone tubing and a syringe pump with a flow rate of 3 μL min^{-1}. Because a Pt electrode was used as the counter electrode, all potentials are reported *vs.* Pt, where 0 V corresponds to approximately 4.02 V *vs.* Li/Li^+. TEM (JEM-3011HR, JEOL) and a charge-coupled device camera (Orius SC1000, Gatan) were employed for the *in situ* electrochemical TEM experiments. The electron irradiation damaged both the silicon nitride window and electrolyte and induced chemical lithiation during TEM observations[47–50]. For stable observations, the flux of the electron beam was <150 e^- nm^{-2} s^{-1} at an accelerating voltage of 300 kV.

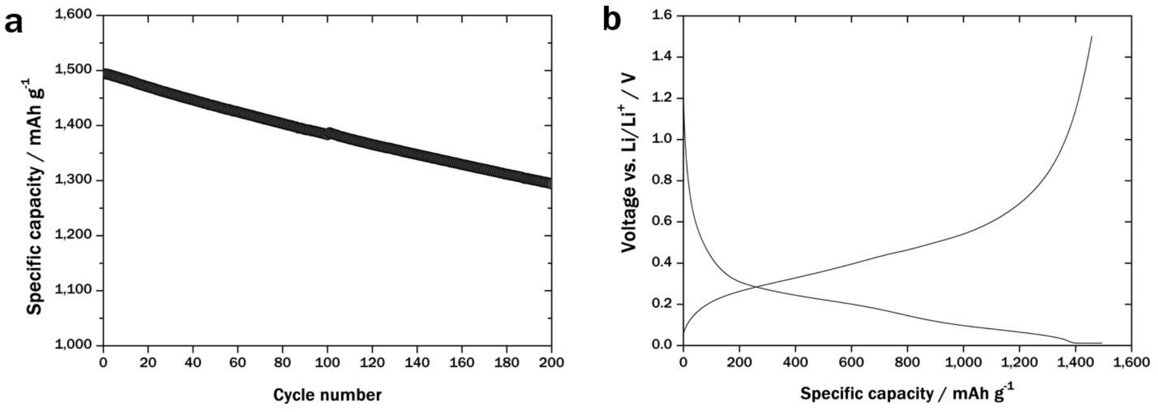

Supplementary Figure 1. Electrochemical properties of surface-treated silicon/carbon (SSC) composite. a Specific capacity retention of SSC at a C-rate of 0.5C. **b** Voltage profile of SSC during the first cycle.

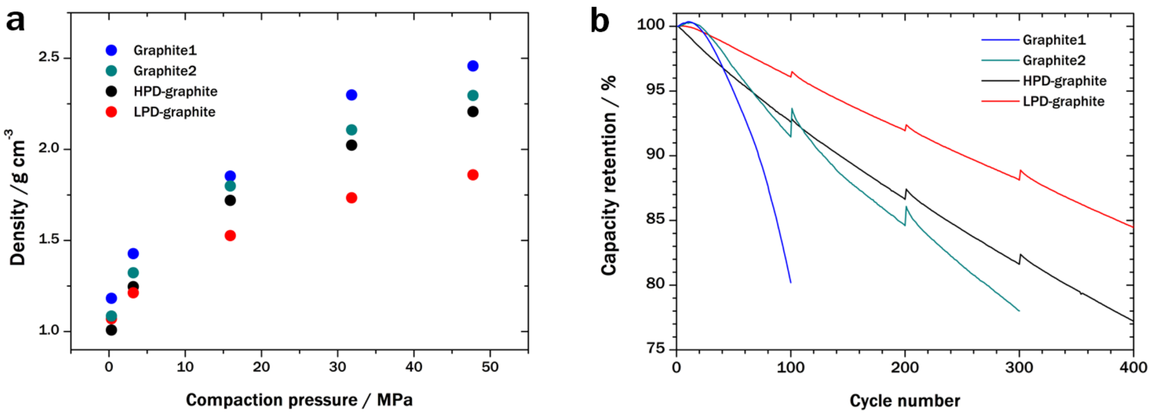

Supplementary Figure 2. Effect of graphite hardness on cycle performance. a Variation in density with compaction pressure for graphite1 (●), graphite2 (●), high pellet density (HPD)-graphite (●), and low pellet density (LPD)-graphite (●). **b** Dependence of physical properties of graphite on the cycling performance of cells using silicon–graphite anodes.

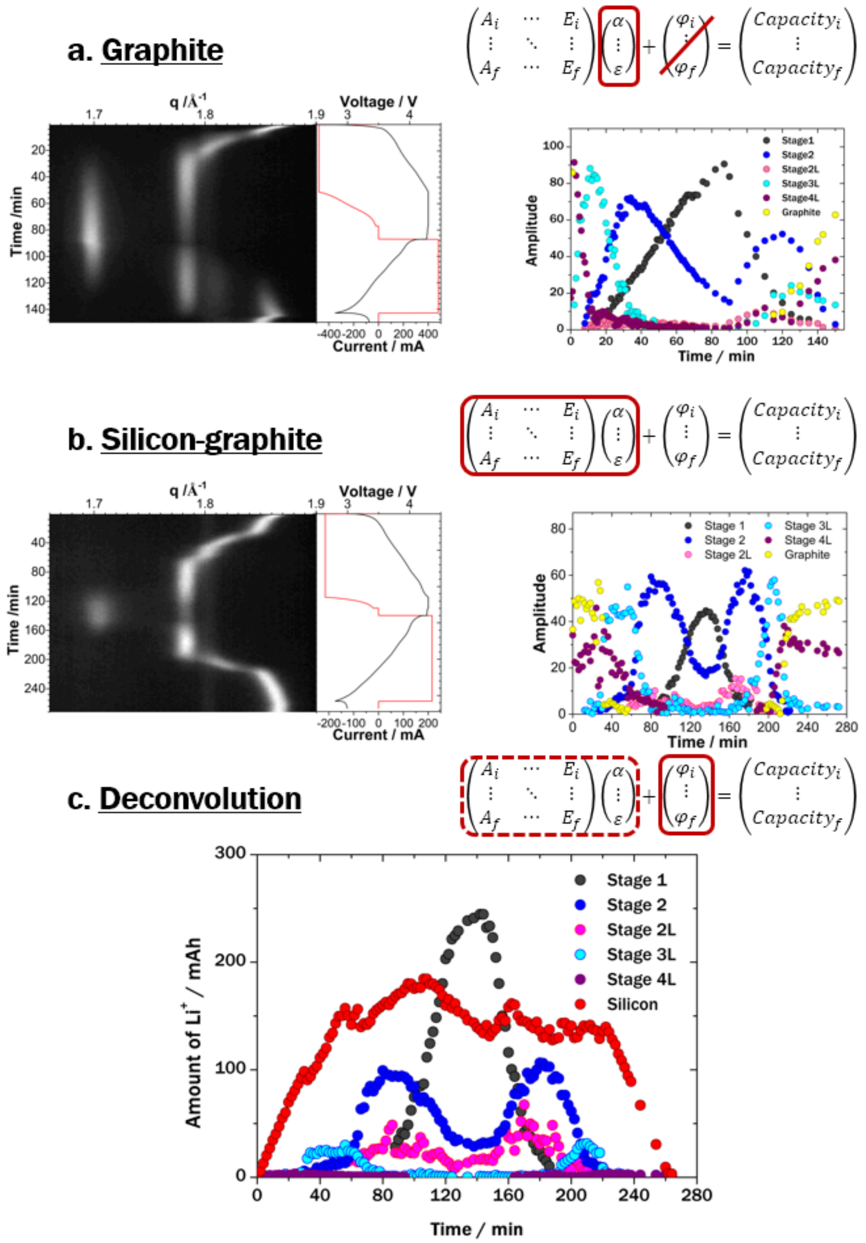

Supplementary Figure 3. Deconvolution of the mixed state of Cell B at 0.5C. a, b X-ray diffraction (XRD) profiles and corresponding voltage and current profiles during battery operation of cells with graphite (Cell A) and silicon–graphite (Cell B) anodes, respectively. The amplitude profiles of the stages of graphite in the anode after the refinement process are shown on the right of each XRD profile. **c** Deconvolution of the mixed state in the anode of Cell B into individual silicon and graphite states.

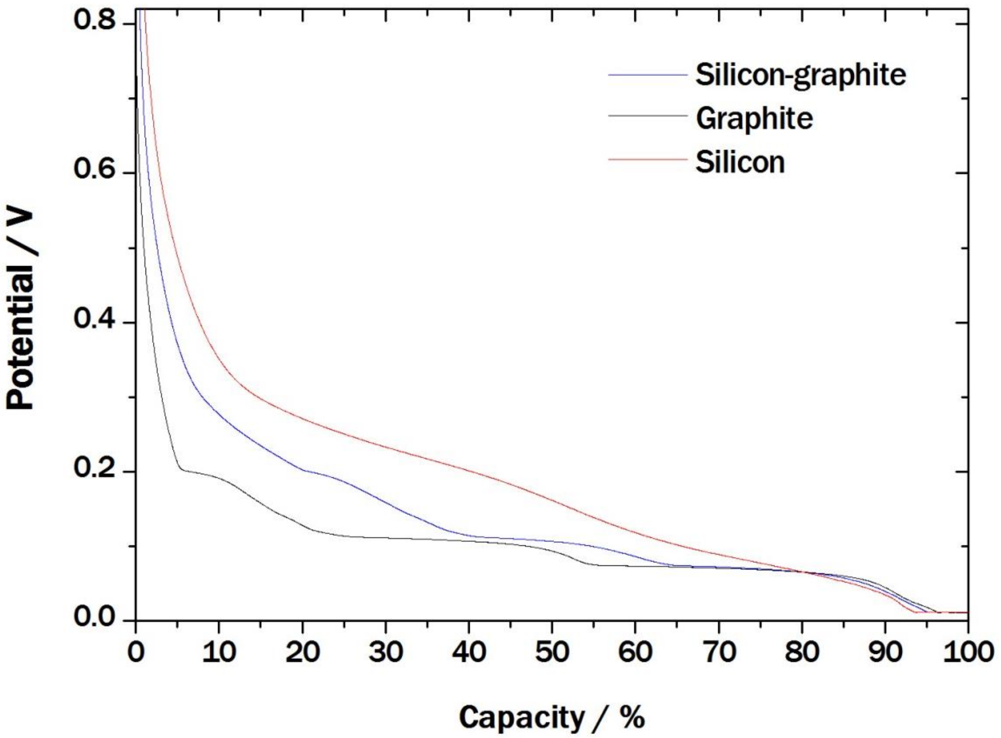

Supplementary Figure 4. Potential profiles of composite anode and individual components. Potentials of graphite, silicon, and silicon–graphite anodes (—, —, and —, respectively) measured at a C-rate of 0.05C by half-cell assessments.

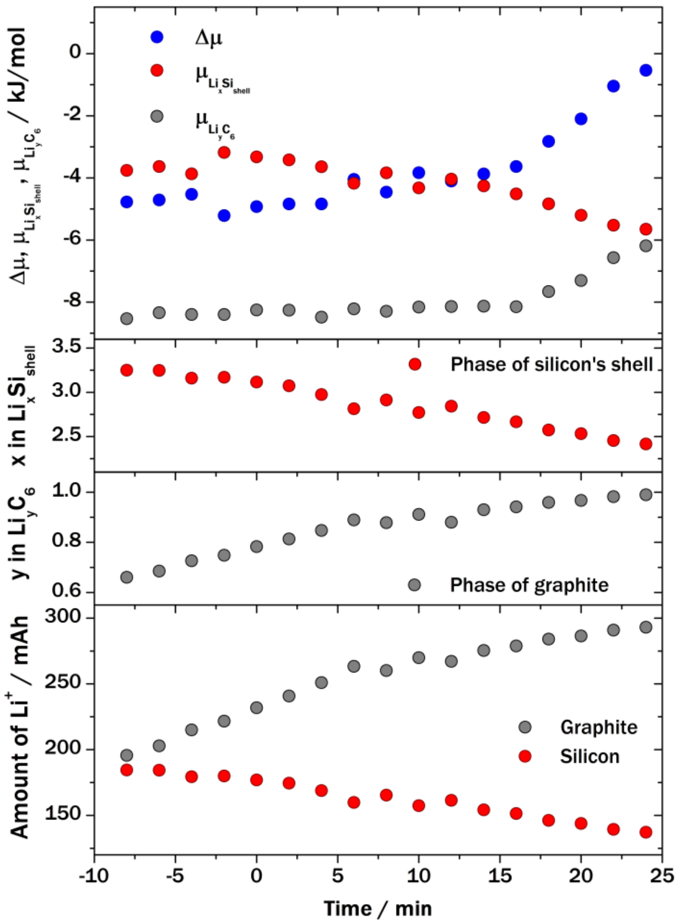

Supplementary Figure 5. Thermodynamic driving force corresponding to the X-ray diffraction (XRD) results during constant coltage (CV) charging and resting of Cell B after operation at 0.5C. The CV charging start time is set to 0 min. After 17 min, the cell was allowed to rest. The upper-most panel shows the chemical potentials of Li_xSi_{shell} and Li_yC_6 as well as their difference ($\Delta\mu$), which approaches zero because of the internal redox reaction between silicon and graphite. In the middle two panels, the phase of Li_yC_6 can be determined using the measured amount of Li^+, and the Li_xSi_{shell} phase can be estimated using the position of the sudden change in the amount of Li^+. The lower panel presents the measured amounts of Li^+ in silicon and graphite.

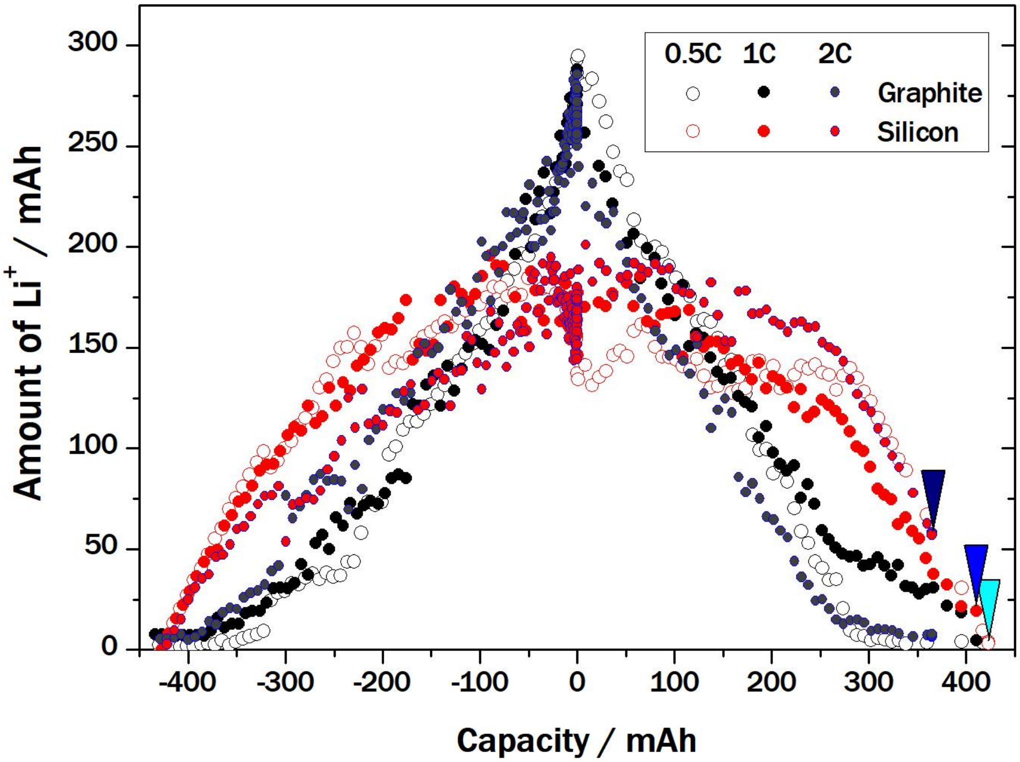

Supplementary Figure 6. Amount of Li^+ in silicon and graphite during the first cycle at various C-rates. The triangles indicate the amount of Li^+ remaining in silicon at the end of the cycle (▼: 57.7 mAh at 2C; ▼: 19.4 mAh at 1C; ▼: 3.2 mAh at 0.5C).

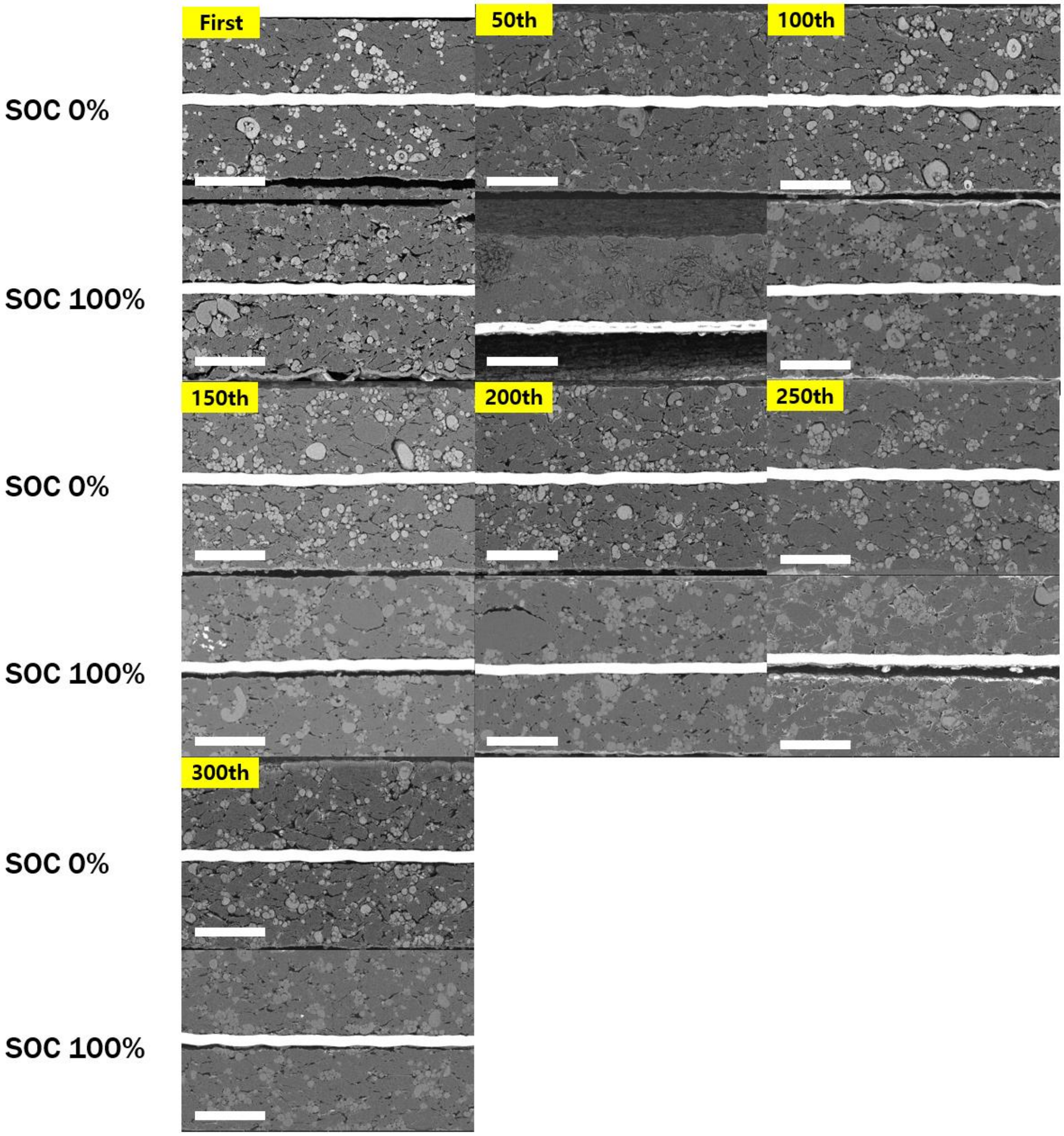

Supplementary Figure 7. Cross-sectional scanning electron microscopy (SEM) images of anodes during cycling. Cross-sectional images at 0% and 100% state-of-charge (SOC) every 50 cycles. These electrodes were obtained after mini-18650 experiments. Scale bars: 50 μm

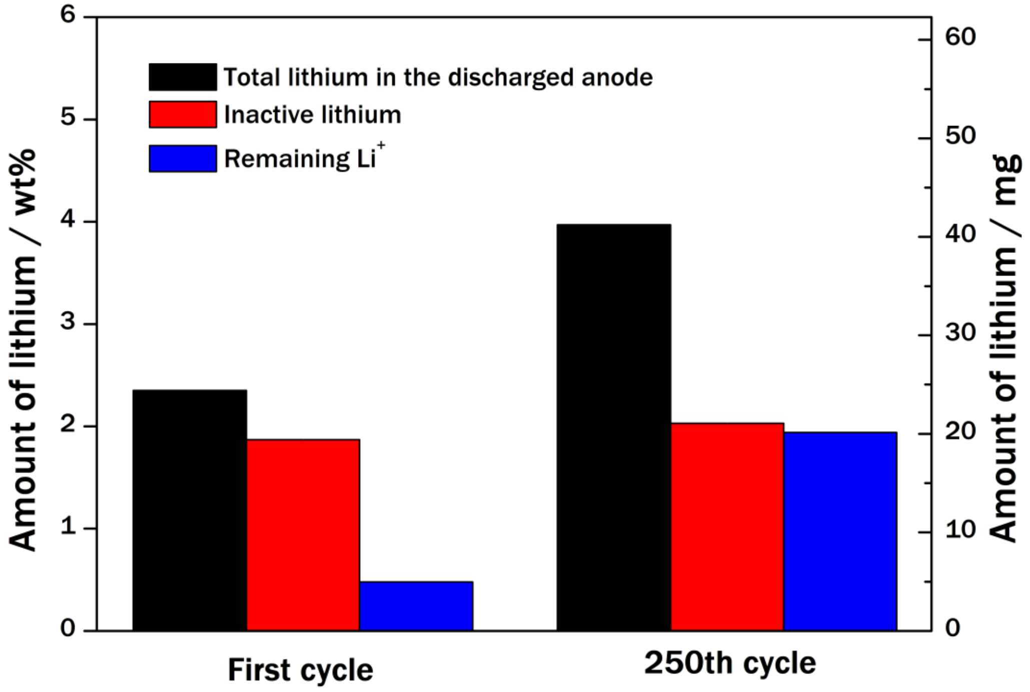

Supplementary Figure 8. Amounts of lithium in anode determined by inductively coupled plasma atomic emission spectroscopy (ICP-AES). Amounts of total lithium, inactive lithium, and remaining Li^+ in the anode of Cell B after the first and 250th cycles.

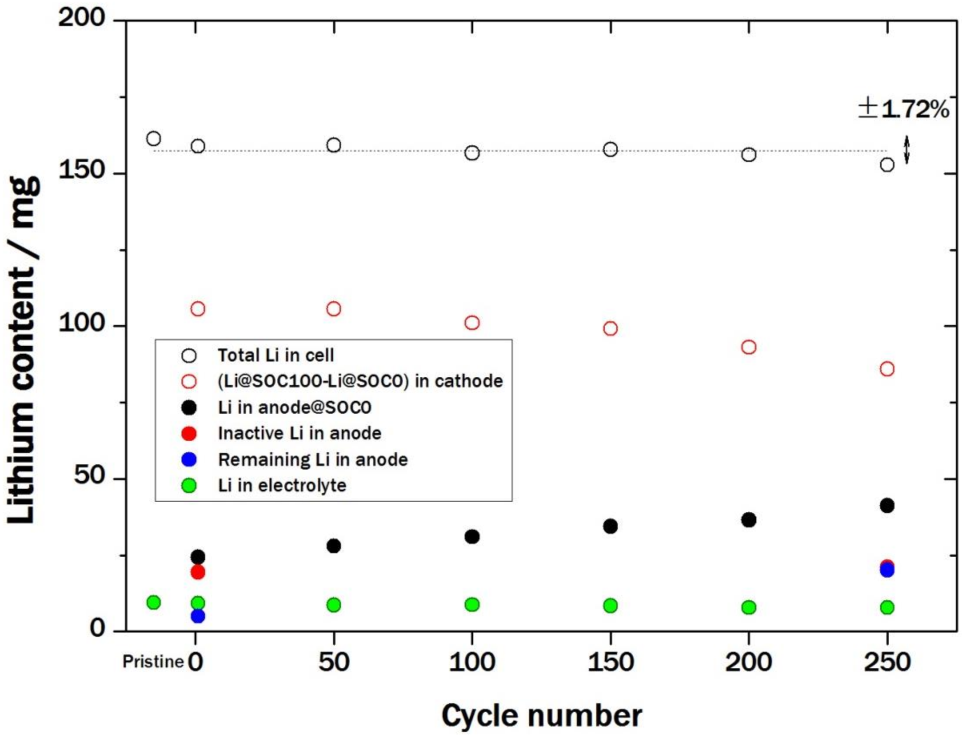

Supplementary Figure 9. Lithium contents in the cell, cathode, anode, and electrolyte. Amounts of lithium in the cell (○), anode (●), and electrolyte (●) of Cell B every 50 cycles. Amounts of active lithium in the cathode (○), inactive lithium in the anode (●), and remaining lithium in the anode (●).

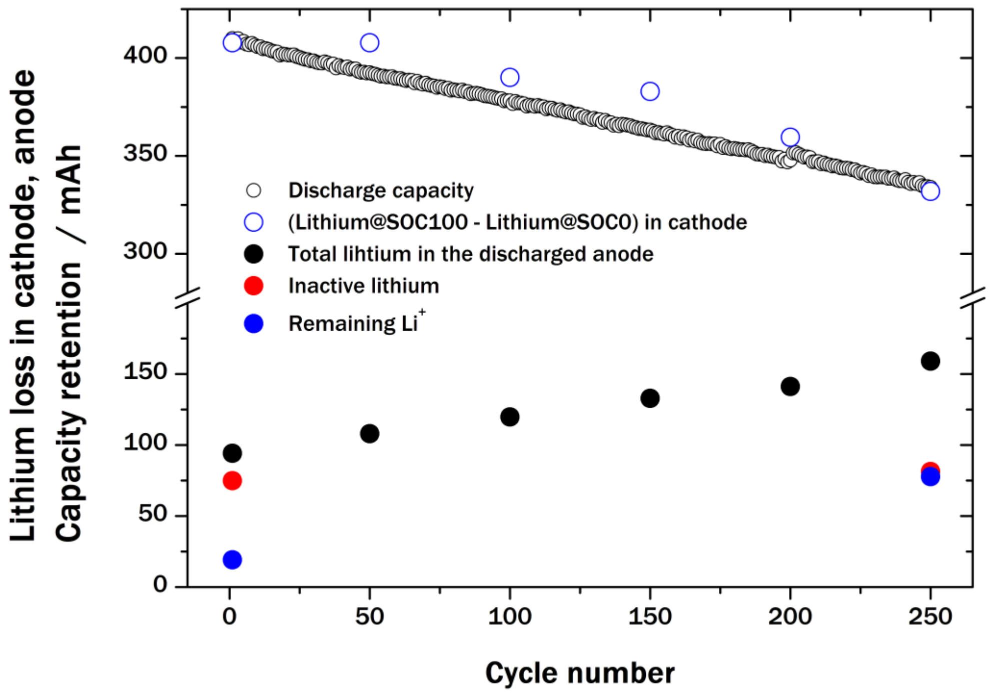

Supplementary Figure 10. Lithium loss in cathode and anode *vs.* capacity retention. Capacity retention of Cell B (○, each cycle), decrease in active lithium in the cathode (○), and lithium loss in the anode (●) originating from inactive lithium (●) and remaining Li^+ (●) in the anode.

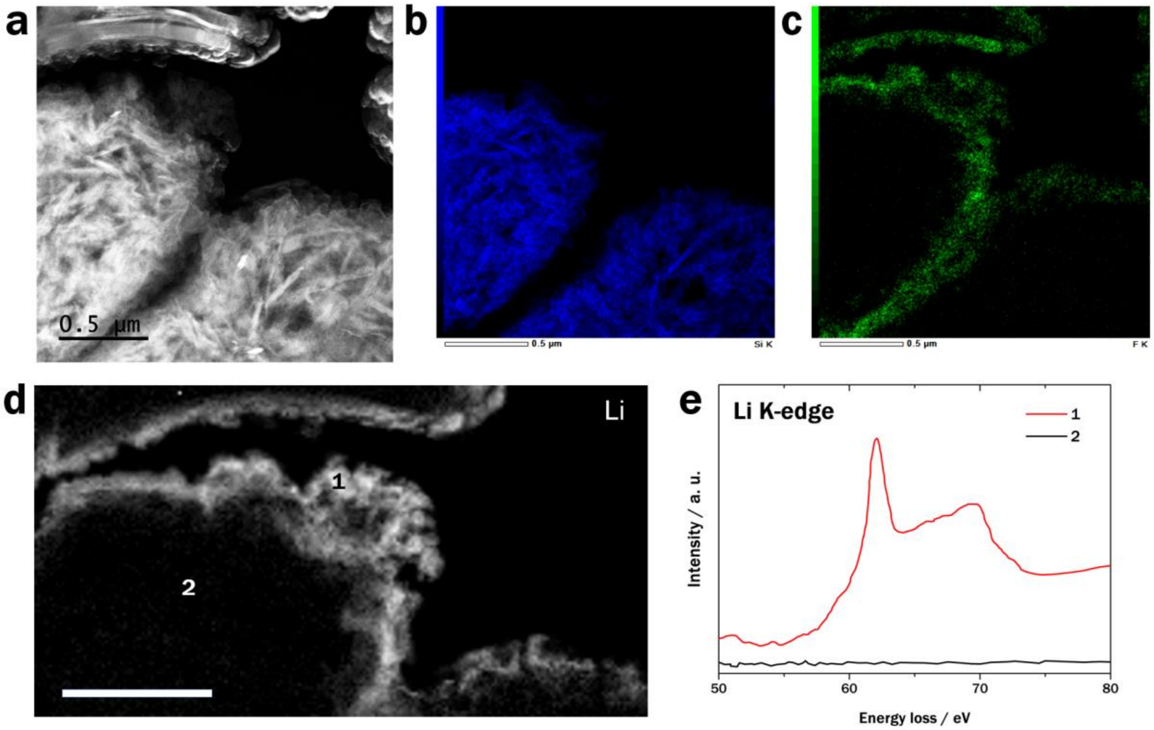

Supplementary Figure 11. Formation of solid electrolyte interphase (SEI) on surface-treated silicon/carbon (SSC) in Cell B after discharge of first cycle. a ADF image of SSC and **b**, **c** corresponding energy dispersive spectroscopy (EDS) mapping images for **b** Si and **c** F. Scale bars: 0.5 μm. **d** Corresponding electron energy loss spectroscopy (EELS) mapping of Li K-edge of SSC (Scale bar: 0.5 μm) and **e** EELS spectra at points 1 and 2 on the EELS mapping image.

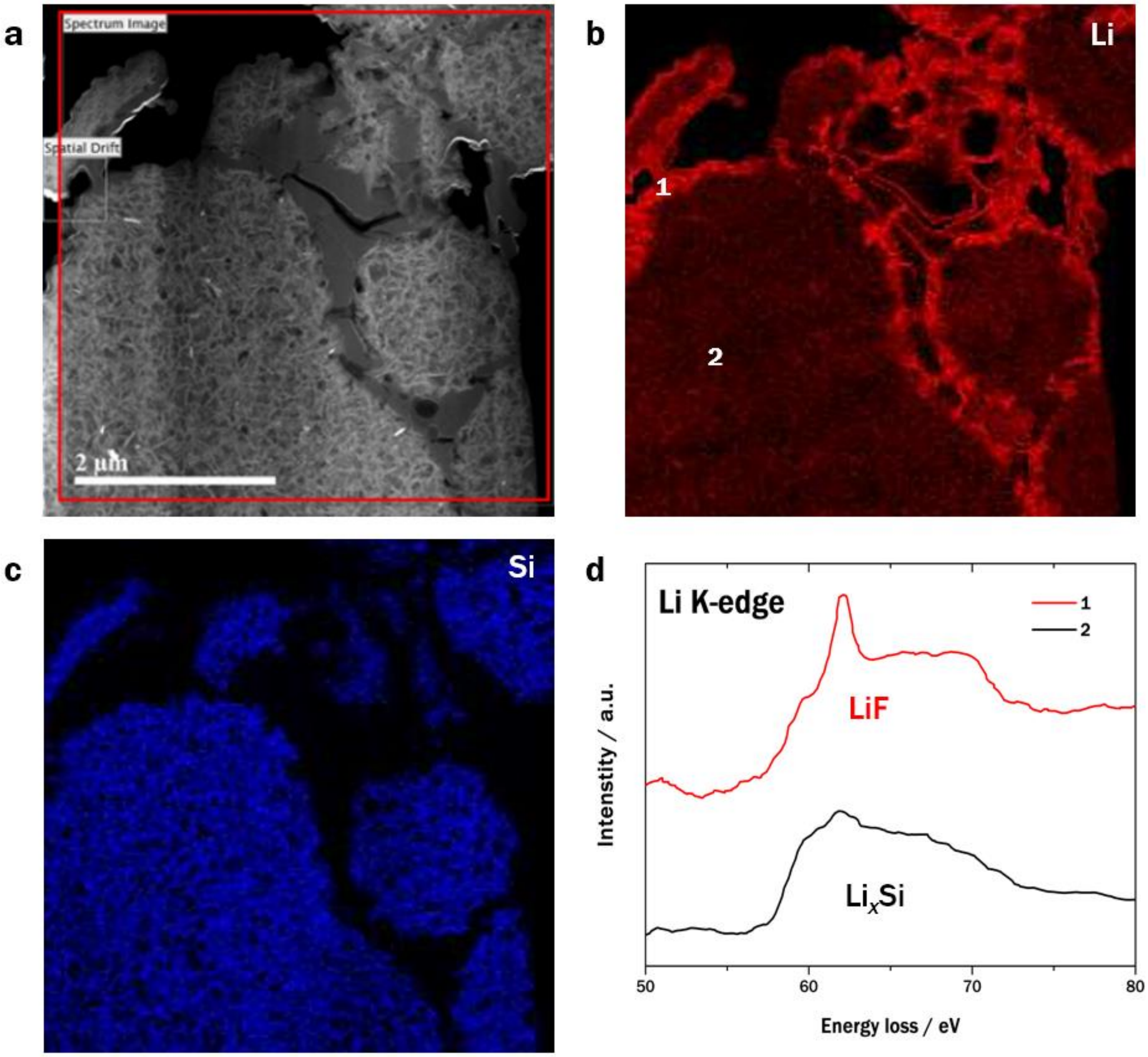

Supplementary Figure 12. Solid electrolyte interphase (SEI) on surface-treated silicon/carbon (SSC) and Li^+ accumulation in SSC of Cell B after discharge of 100th cycle. a ADF image and **b**, **c** corresponding electron energy loss spectroscopy (EELS) mappings of **b** Li K- and **c** Si L-edge of SSC. Scale bars: 2 μm. **d** Li K-edge EELS spectra at points 1 and 2 on the Li K-edge mapping image.

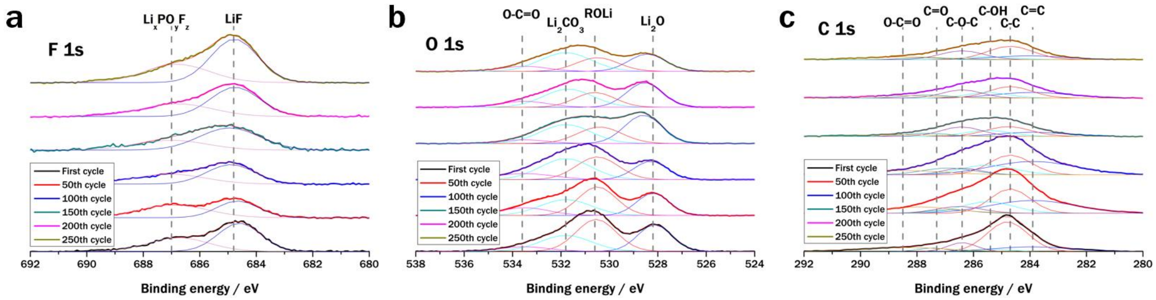

Supplementary Figure 13. Solid electrolyte interphase (SEI) chemical composition on cycled anodes of Cell B. X-ray photoelectron spectroscopy (XPS) measurements of anodes of Cell B every 50 cycles. **a** *F 1s*, **b** *O 1s*, and **c** *C 1s* spectra.

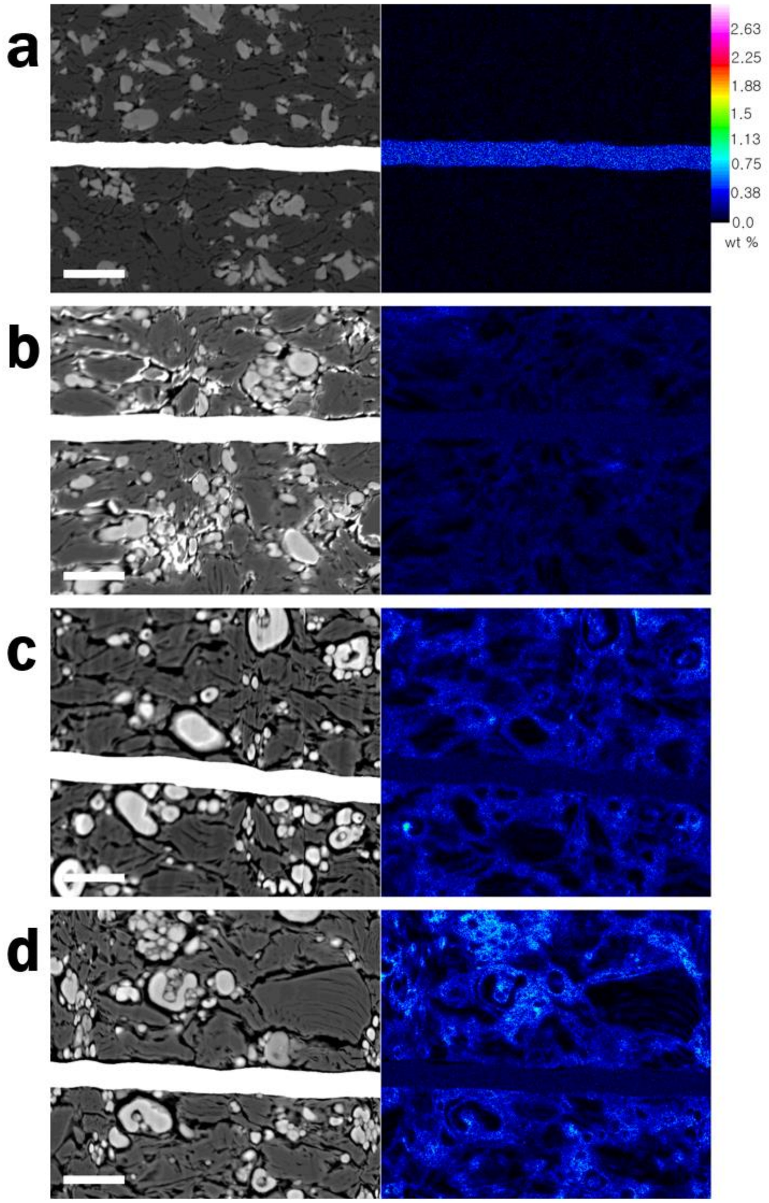

Supplementary Figure 14. Electron probe micro-analysis (EPMA) characterisation of solid electrolyte interphase (SEI) in Cell B. Elemental analysis of the cross-sectioned anodes in **a** pristine state and at **b** first, **c** 100th, and **d** 200th cycles using EPMA. Left: Cathodoluminescence (CL) images (Scale bars: 20 μm). Right: Elemental distribution maps of P (Colour scale: concentration of P (wt%) in the anode).

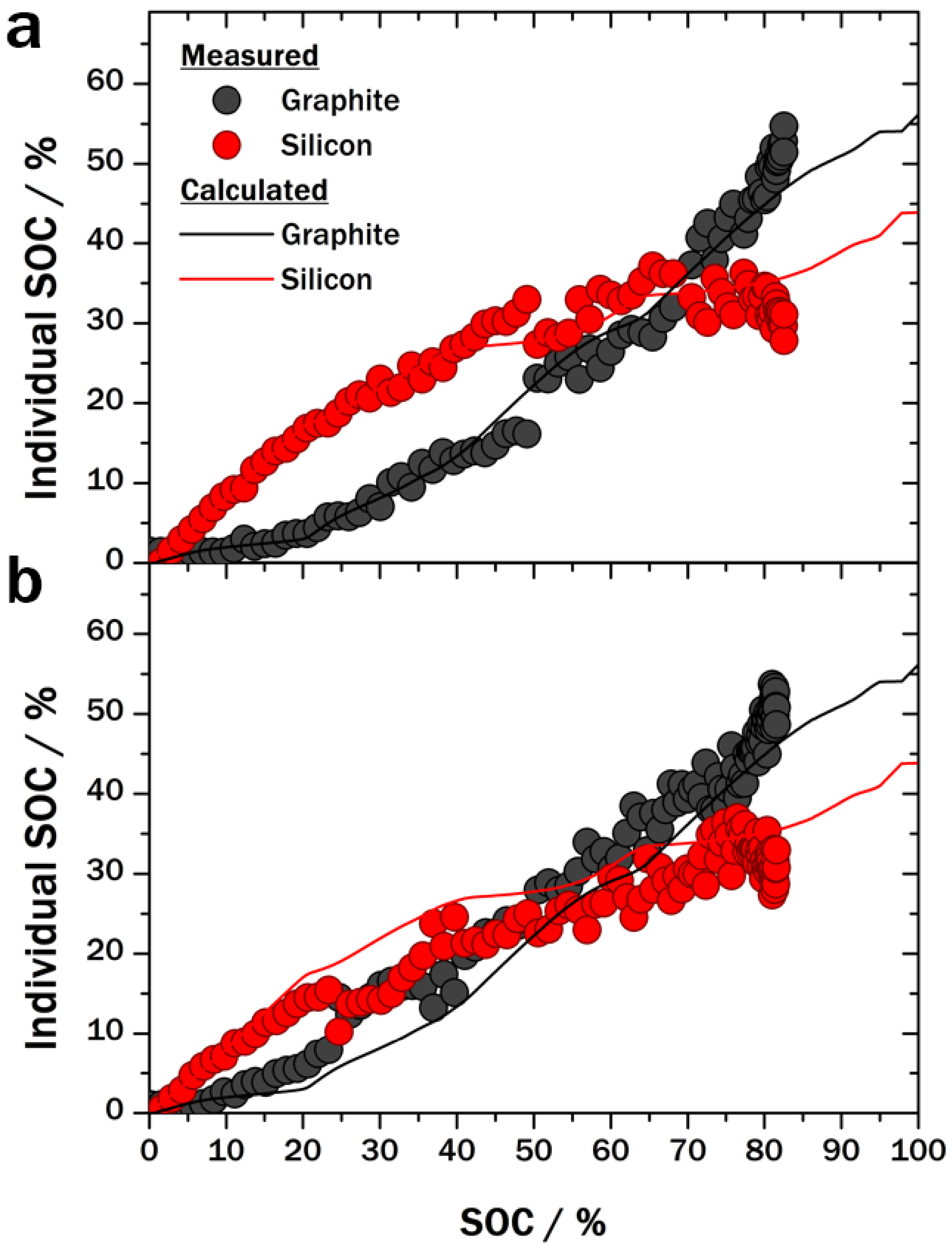

Supplementary Figure 15. States-of-charge (SOCs) in individual materials during charging at C-rates of 1C and 2C at the first cycle. Comparison of experimental and calculated individual SOCs in the anode of Cell B during charging at **a** 1C and **b** 2C. The calculated lines were determined based on half-cell experiments at a low C-rate of 0.05C. ● and ● correspond to data from full-cell experiments.

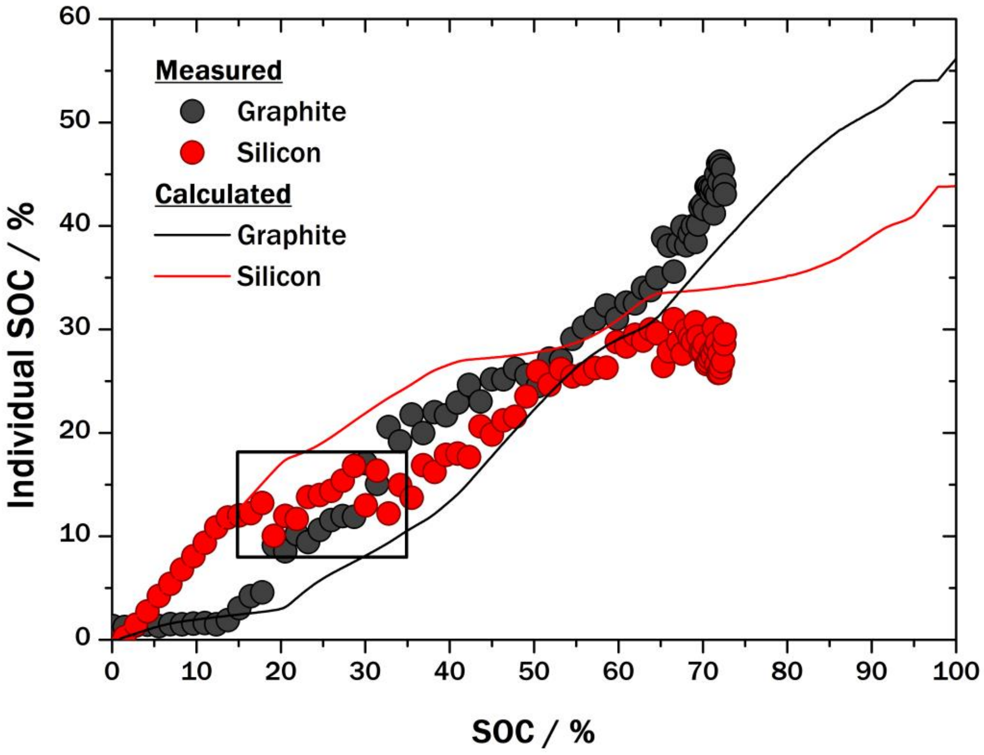

Supplementary Figure 16. States-of-charge (SOCs) in individual materials at the 250th cycle. Individual SOCs of graphite and silicon (● and ●, respectively) corresponding to the charging process of the 250th cycle in Fig. 2b. The delay region for Li^+ transfer to silicon is indicated by the black box.

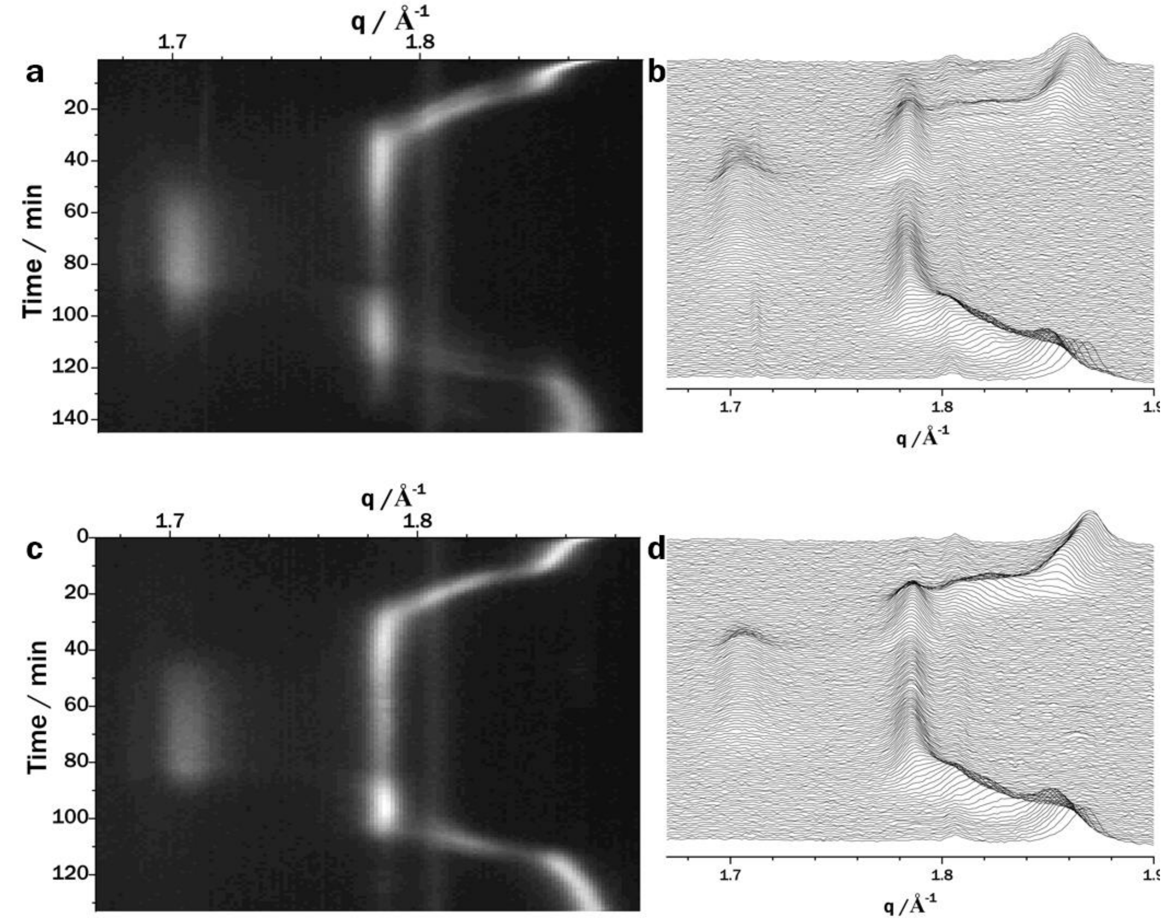

Supplementary Figure 17. Raw X-ray diffraction (XRD) data for Cell B. XRD profiles of graphite at the **a, b** first and **c, d** 250th cycles of Cell B.

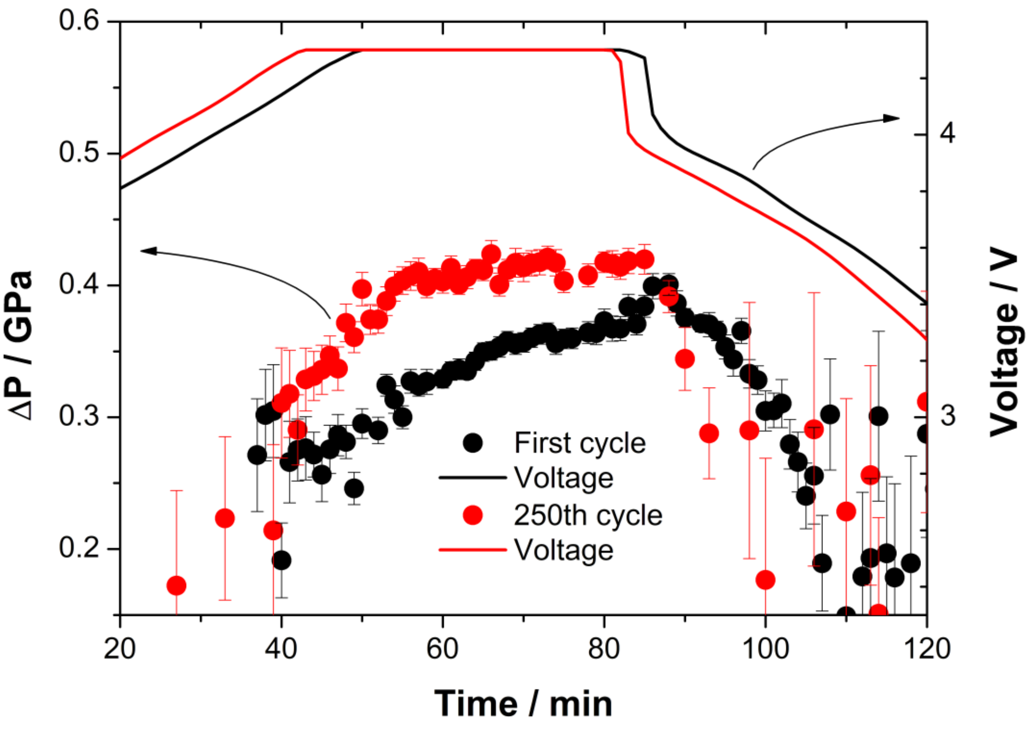

Supplementary Figure 18. Evolution of pressure on graphite owing to volume expansion of Li_xSi in Cell B. Calculated pressure (ΔP) on graphite at the first and 250th cycles (● and ●, respectively) and corresponding voltage profiles (— and —, respectively). The pressures were calculated from the X-ray diffraction (XRD) data in Supplementary Fig. 17. The errors in evolution of pressure on graphite are quite large owing to small intensities of stage 1 peaks when the stage 1 partition in graphite is small.

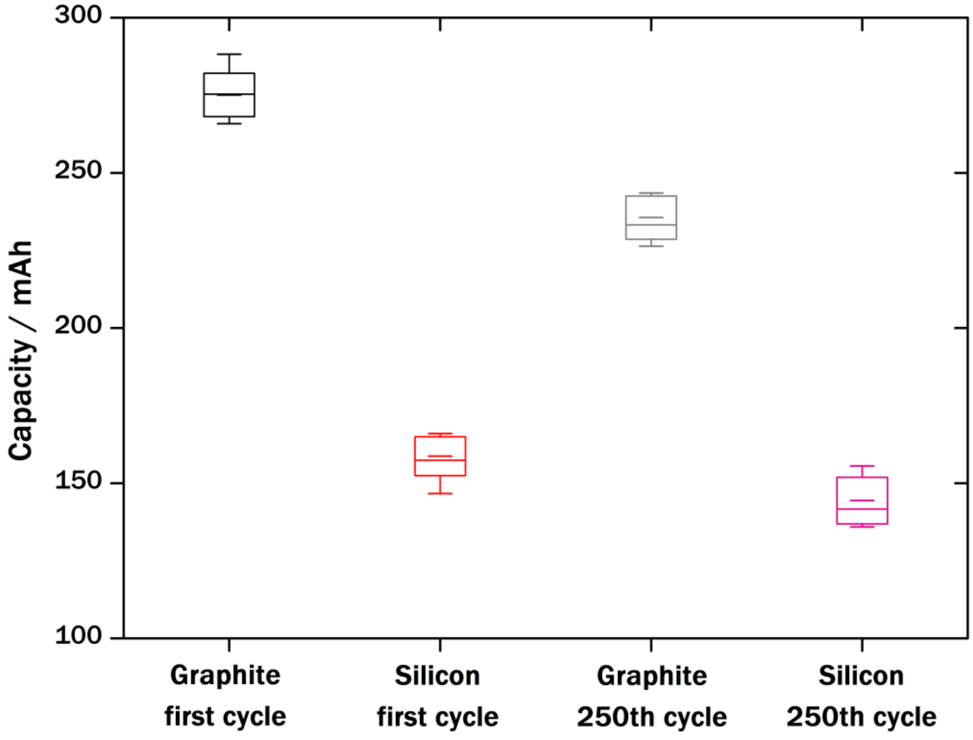

Supplementary Figure 19. Charge capacities of graphite and silicon at the first and 250th cycles. Box plots of measured 10 points in Fig. 2b at the end of charging (rest step). Horizontal lines going through the boxes denote medians; boxes and whiskers denote mean (short horizontal line in box) ± SD and 0th and 100th percentiles, respectively.

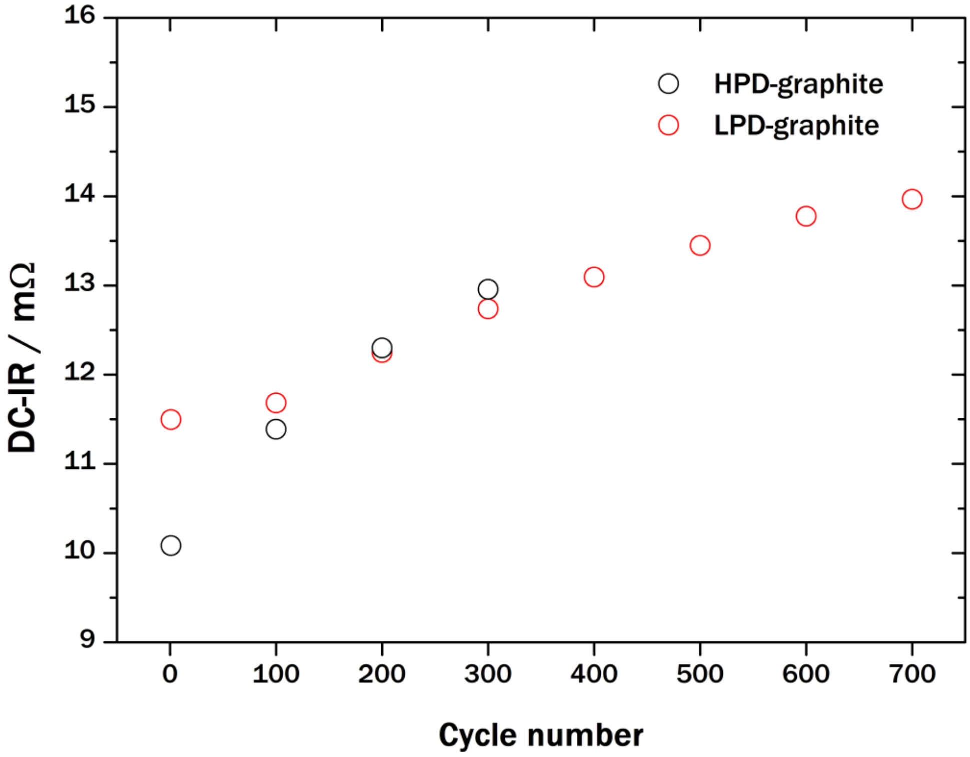

Supplementary Figure 20. Direct current–internal resistance (DC–IR) characteristics of prismatic cells with a capacity of 8.7 Ah. Cells fabricated with anode containing high pellet density (HPD)-graphite (○) and low pellet density (LPD)-graphite (○). The DC–IR increases with long-term cycling.

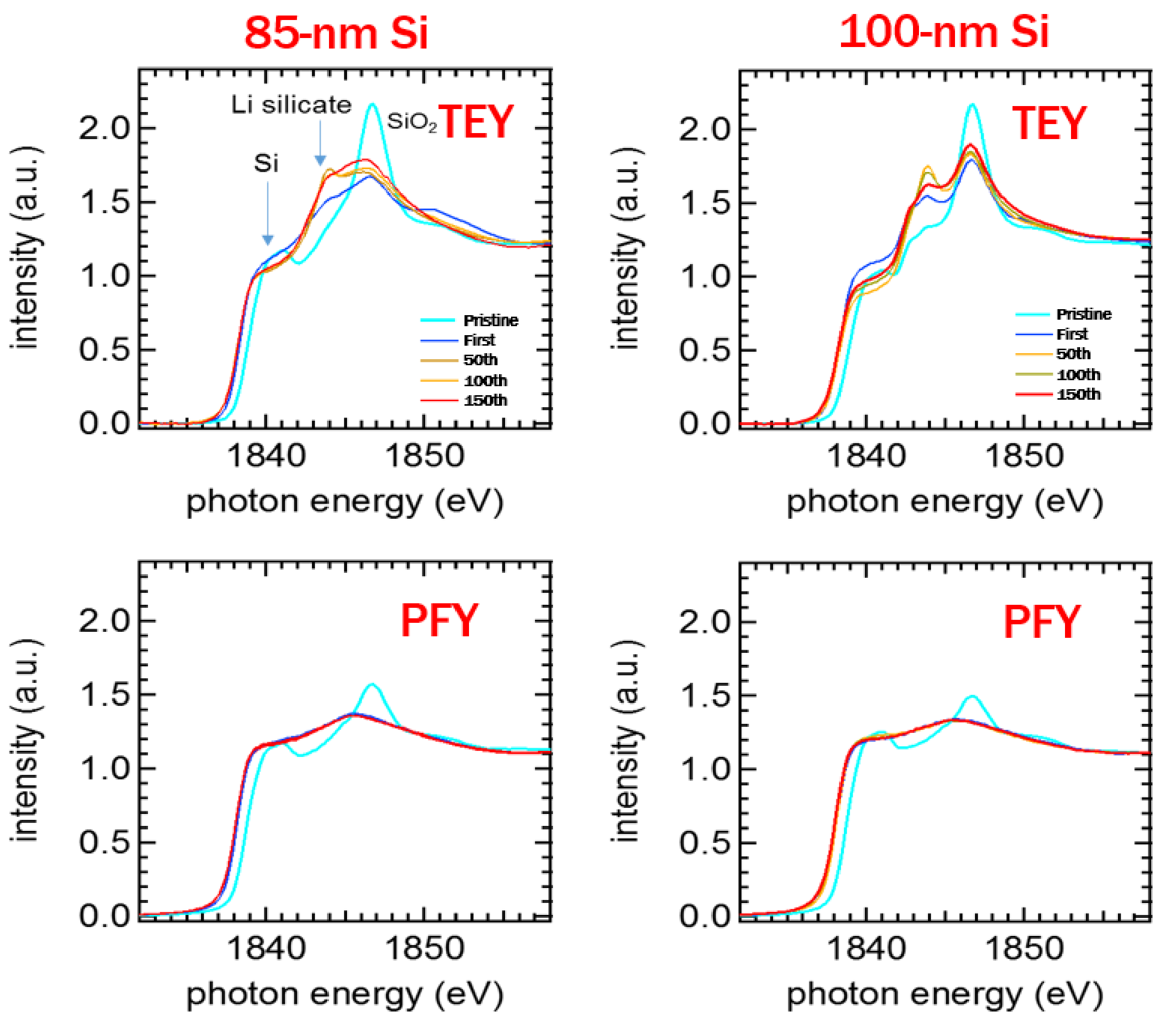

Supplementary Figure 21. Initial silicon oxide content measured by total-electron-yield (TEY)- and partial-fluorescence-yield (PFY)-X-ray absorption near edge structure (XANES) spectroscopy. Initial silicon oxide content in silicon particles with long-axes of 85 and 100 nm (85- and 100-nm Si, respectively) is removed during formation stage.

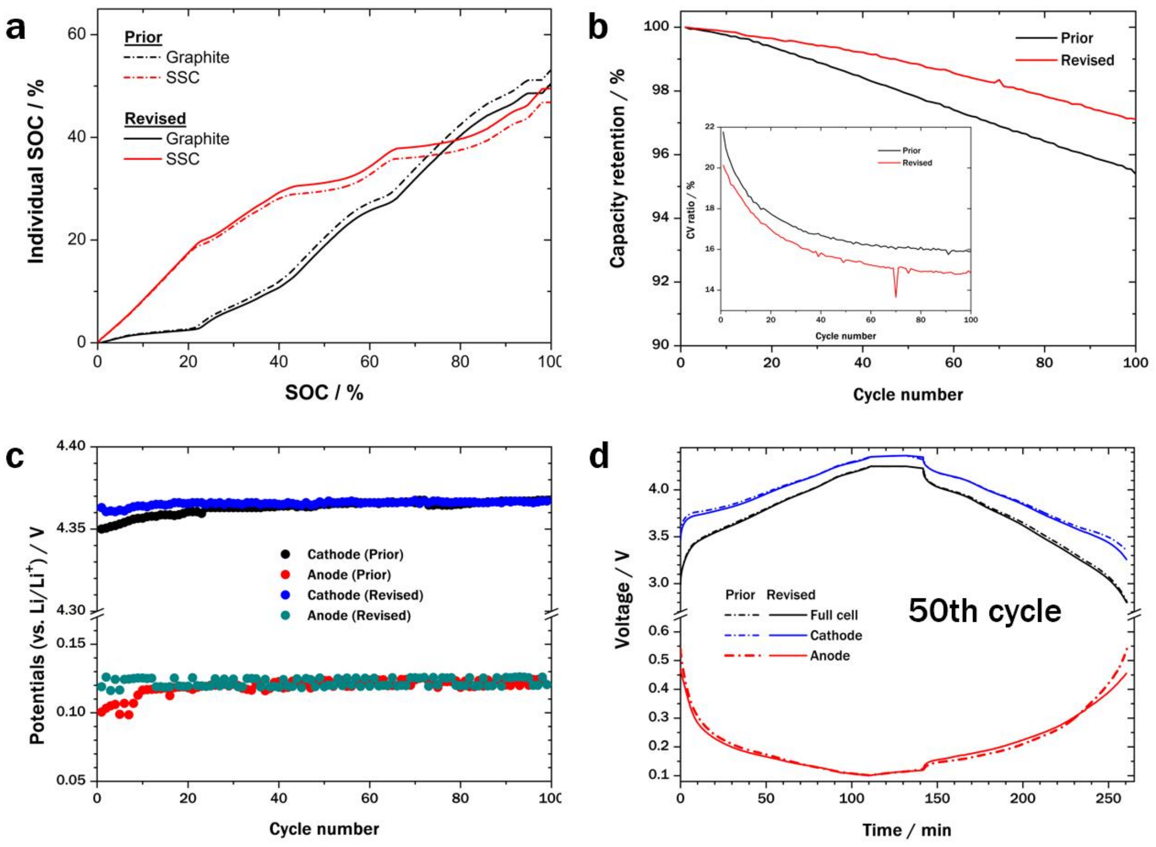

Supplementary Figure 22. Change in electrode design. a Comparison of calculated individual SOCs for prior and revised designs. **b** Improved performance of revised cell in terms of capacity retention and constant voltage (CV) ratio. **c** Cathode and anode potential at the end of charging stage of various cycles. **d** Potential profiles of a full cell and individual electrodes at the 50th cycle.

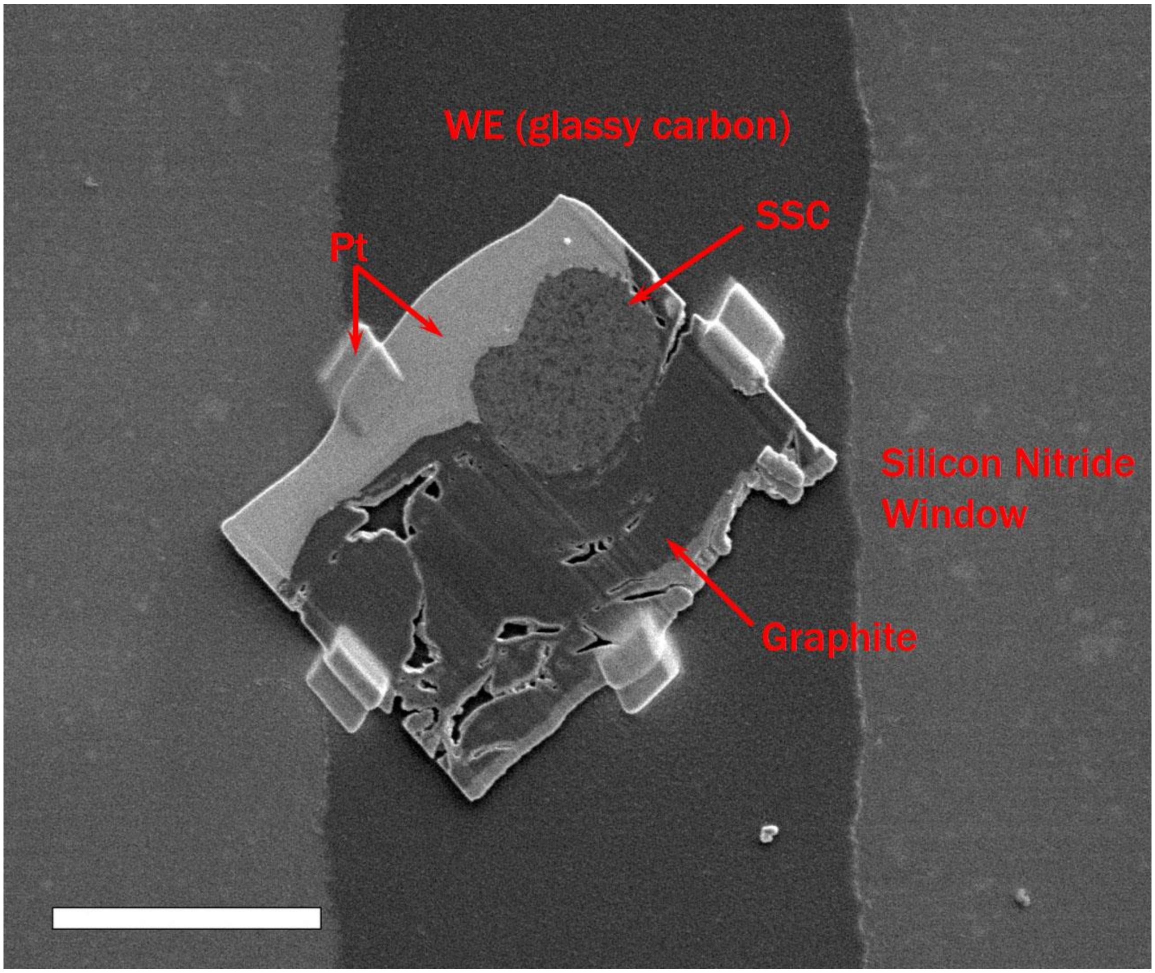

Supplementary Figure 23. Specimen for *in situ* electrochemical TEM. SEM image of the TEM specimen on the working electrode (WE). SSC: Surface-treated silicon/carbon composite. Scale bar: 5 μm.

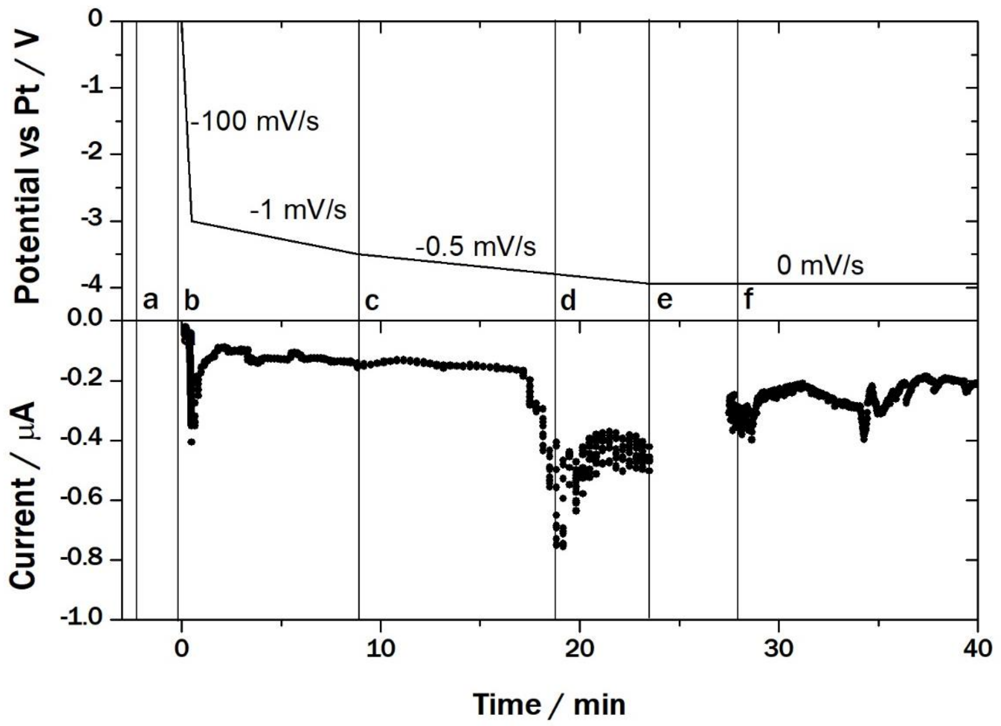

Supplementary Figure 24. Potential waveform used to observe lithiation in the silicon–graphite anode and corresponding current. TEM micrographs were obtained at points a–f.

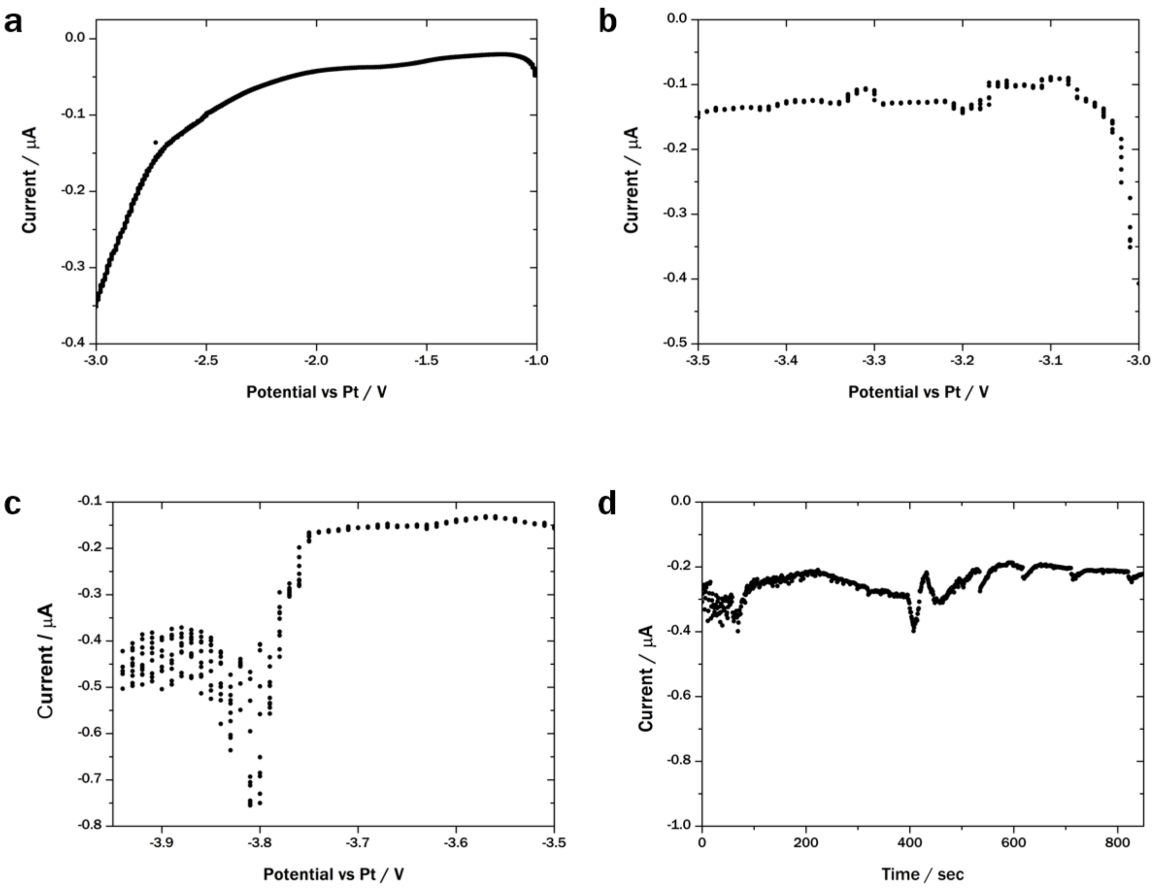

Supplementary Figure 25. Electrochemical measurement during *in situ* electrochemical TEM. Linear sweep voltammograms from **a** −1 to −3 V with a ramp rate of −100 mV s^{-1}; **b** −3 to −3.5 V with a ramp rate of −1 mV s^{-1}; and **c** −3.5 to −3.94 V with a ramp rate of −0.5 mV s^{-1}. **d** Chronoamperogram at constant potential of −3.94 V.

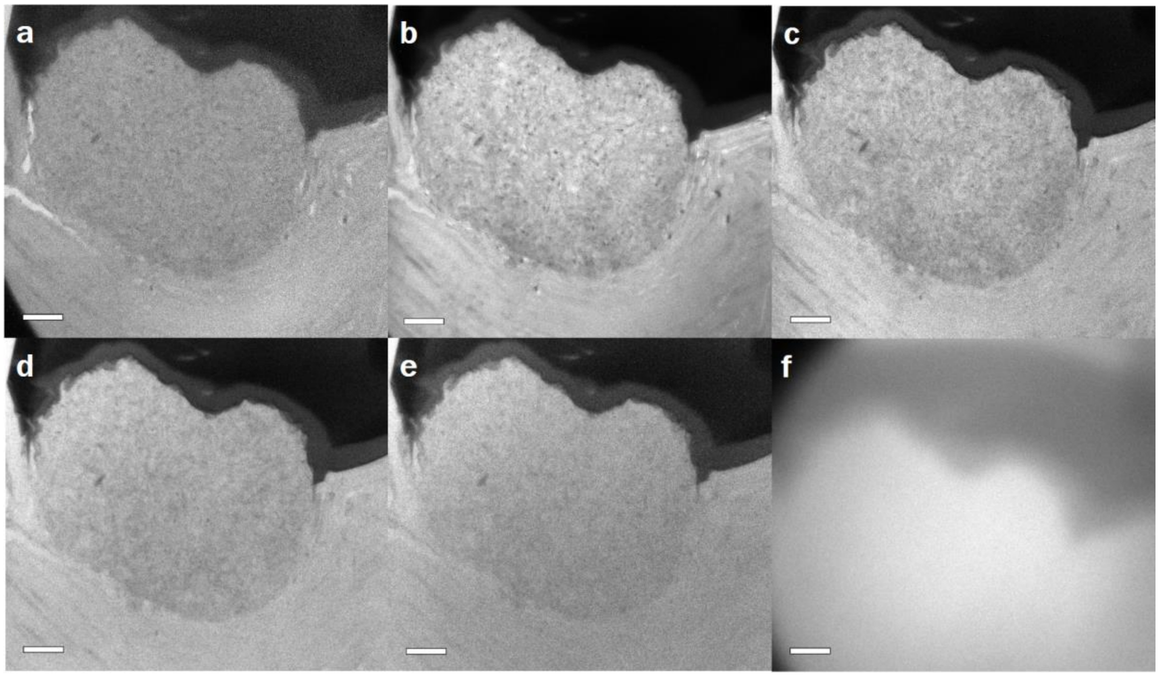

Supplementary Figure 26. ***In situ*** **electrochemical TEM characterisation of silicon–graphite during lithiation. a** Cross-sectional TEM image of graphite before filling with electrolyte. **b**–**e** TEM images of silicon–graphite during lithiation from 0 to −3.94 V *vs.* Pt. **b**: 0 s; **c**: 8 min 50 s, corresponding to −3.5 V; **d**: 18 min 50 s, corresponding to −3.8 V; **e**: 23 min 30 s, corresponding to −3.94 V; and **f**: 28 min during chronoamperometry at a constant potential of −3.94 V. Scale bars: 500 nm.

Supplementary Table 1. Lithium contents in the cathode, anode, and electrolyte of Cell B.

	Cathode /wt%	Cathode /mg	Cathode /mAh	Anode /wt%	Anode /mg	Anode /mAh	Electrolyte /wt%	Electrolyte /mg
**Pristine**	5.949	151.82	586.55				0.73	9.42
**First cycle (0% SOC)**	4.909	125.26	483.95	2.35	24.36	94.12	0.72	9.26
**Inactive Li**				1.87	19.39	74.90		
**First cycle (100% SOC)**	0.772	19.70	76.10	11.78	122.15	471.92	0.69	8.95
**50th cycle (0% SOC)**	4.805	122.61	473.69	2.70	27.94	107.94	0.67	8.65
**50th cycle (100% SOC)**	0.668	17.04	65.84	11.86	122.82	474.49	0.66	8.49
**100th cycle (0% SOC)**	4.579	116.85	451.46	2.99	31.01	119.81	0.68	8.80
**100th cycle (100% SOC)**	0.622	15.89	61.37	11.79	122.19	472.07	0.66	8.49
**150th cycle (0% SOC)**	4.509	115.06	444.54	3.32	34.39	132.87	0.65	8.34
**150th cycle (100% SOC)**	0.625	15.95	61.60	12.15	125.99	486.75	0.60	7.72
**200th cycle (0% SOC)**	4.377	111.68	431.48	3.51	36.53	141.13	0.61	7.87
**200th cycle (100% SOC)**	0.729	18.62	71.92	11.52	119.79	462.81	0.57	7.41
**250th cycle (0% SOC)**	4.065	103.74	400.79	3.97	41.16	159.01	0.61	7.87
**Inactive Li**				2.03	21.05	81.31		
**250th cycle (100% SOC)**	0.698	17.82	68.85	12.08	125.26	483.92	0.61	7.87

Supplementary Table 2. Information on cells.

Type or test	Capacity / mAh	Areal Capacity / mAh cm^{-2}	*n*/*p*	Operating Condition / V	Cathode	Anode	Electrolyte
Cell A	480	4.4	1.08	2.8–4.3	$Li_{1.0}Ni_{0.88}Co_{0.08}Mn_{0.04}O_2$	HPD-graphite	1.15 M $LiPF_6$ in FEC; EC; EMC; DMC
Cell B	430	4.4	1.03	2.8–4.3	$Li_{1.0}Ni_{0.88}Co_{0.08}Mn_{0.04}O_2$	SSC–HPD-graphite	1.15 M $LiPF_6$ in FEC; EC; EMC; DMC
Cell C	630	6.0	1.03	2.8–4.25	$Li_{1.0}Ni_{0.88}Co_{0.08}Mn_{0.04}O_2$	SSC–HPD-graphite	1.15 M $LiPF_6$ in FEC; EC; EMC; DMC
Cell D	630	6.0	1.03	2.8–4.25	$Li_{1.0}Ni_{0.88}Co_{0.08}Mn_{0.04}O_2$	SSC–LPD-graphite	1.15 M $LiPF_6$ in FEC; EC; EMC; DMC
Prismatic cell	8700	6.0	1.03	2.8–4.25	$Li_{1.0}Ni_{0.88}Co_{0.08}Mn_{0.04}O_2$	SSC–LPD-graphite	1.15 M $LiPF_6$ in FEC; EC; EMC; DMC
In situ force measurements (pouch cell)	1500	4.4	1.08	2.8–4.25	$Li_{1.0}Ni_{0.88}Co_{0.08}Mn_{0.04}O_2$	HPD-graphite	1.15 M $LiPF_6$ in FEC; EC; EMC; DMC
In situ force measurements (pouch cell)	1500	6.0	1.03	2.8–4.25	$Li_{1.0}Ni_{0.88}Co_{0.08}Mn_{0.04}O_2$	SSC–HPD-graphite	1.15 M $LiPF_6$ in FEC; EC; EMC; DMC
Mini-18650 cell	610	3.4	1.03	2.8–4.2	$Li_{1.0}Ni_{0.6}Co_{0.2}Mn_{0.2}O_2$ (80%); $Li_{1.0}Ni_{0.8}Co_{0.1}Al_{0.1}O_2$ (20%)	SSC–HPD-graphite	1.15 M $LiPF_6$ in FEC; EC; EMC; DMC
In situ thickness measurements (pouch cell)	81	3.4	1.08	2.8–4.2	$Li_{1.0}Ni_{0.6}Co_{0.2}Mn_{0.2}O_2$ (80%); $Li_{1.0}Ni_{0.8}Co_{0.1}Al_{0.1}O_2$ (20%)	HPD-graphite	1.15 M $LiPF_6$ in EC; EMC; DMC
In situ thickness measurements (pouch cell)	72	3.4	1.03	2.8–4.2	$Li_{1.0}Ni_{0.6}Co_{0.2}Mn_{0.2}O_2$ (80%); $Li_{1.0}Ni_{0.8}Co_{0.1}Al_{0.1}O_2$ (20%)	SSC–HPD-graphite	1.15 M $LiPF_6$ in EC; EMC; DMC

Supplementary References

1. Hryha, E., Zubko, P., Dudrová, E., Pešek, L. & Bengtsson, S. An application of universal hardness test to metal powder particles. *J. Mater. Process. Technol.* **209,** 2377–2385 (2009).

2. Ohzuku, T., Iwakoshi, Y. & Sawai, K. Formation of lithium-graphite intercalation compounds in nonaqueous electrolytes and their application as a negative electrode for a lithium ion (shuttlecock) cell. *J. Electrochem. Soc.* **140,** 2490–2498 (1993).

3. Billaud, D. & Henry, F. X. Structural studies of the stage III lithium–graphite intercalation compound. *Solid State Commun.* **124,** 299–304 (2002).

4. Billaud, D., Henry, F. X., Lelaurain, M. & Willmann, P. Revisited structures of dense and dilute stage II lithium-graphite intercalation compounds. *J. Phys. Chem. Solids* **57,** 775–781 (1996).

5. Woo, K. C. *et al.* Effect of in-plane density on the structural and elastic properties of graphite intercalation compounds. *Phys. Rev. Lett.* **50,** 182–185 (1983).

6. Guerard, D. & Herold, A. Intercalation of lithium into graphite and other carbons. *Carbon* **13,** 337–345 (1975).

7. Heß, M. & Novák, P. Shrinking annuli mechanism and stage-dependent rate capability of thin-layer graphite electrodes for lithium-ion batteries. *Electrochim. Acta* **106,** 149–158 (2013).

8. Penrose, R. On best approximate solution of linear matrix equations. *Math. Proc. Cambridge Philos. Soc.* **52,** 17–19 (1956).

9. Kganyago, K. R. & Ngoepe, P. E. Structural and electronic properties of lithium intercalated graphite LiC_6. *Phys. Rev. B* **68,** 205111 (2003).

10. Liu, X. H. *et al.* Self-limiting lithiation in silicon nanowires. *ACS Nano* **7,** 1495–1503 (2013).

11. Sethuraman, V. A., Srinivasan, V. & Newman, J. Analysis of electrochemical lithiation and delithiation kinetics in silicon. *J. Electrochem. Soc.* **160,** A394–A403 (2013).

12. Barin, I. *Thermochemical Data of Pure Substances,* 3rd ed. (VCH, 1995).

13. Aydinol, M. K., Kohan, A. F., Ceder, G., Cho, K. & Joannopoulos, J. *Ab initio* study of lithium intercalation in metal oxides and metal dichalcogenides. *Phys. Rev. B* **56,** 1354–1365 (1997).

14. Wen, C. J. & Huggins, R. A. Chemical diffusion in intermediate phases in the lithium-silicon system. *J. Solid State Chem.* **37,** 271–278 (1981).

15. Liang, S.-M. *et al.* Thermodynamics of Li-Si and Li-Si-H phase diagrams applied to hydrogen absorption and Li-ion batteries. *Intermetallics* **81,** 32–46 (2017).

16. Tipton, W. W., Bealing, C. R., Mathew, K. & Hennig, R. G. Structures, phase stabilities, and electrical potentials of Li-Si battery anode materials. *Phys. Rev. B* **87,** 184114 (2013).

17. Reynier, Y., Yazami, R. & Fultz, B. The entropy and enthalpy of lithium intercalation into graphite. *J. Power Sources* **119–121,** 850–855 (2003).

18. Pietsch, P. *et al.* Quantifying microstructural dynamics and electrochemical activity of graphite and silicon-graphite lithium ion battery anodes. *Nat. Commun.* **7,** 12909 (2016).

19. Bazant, M. Z. Theory of chemical kinetics and charge transfer based on nonequilibrium thermodynamics. *Acc. Chem. Res.* **46,** 1144–1160 (2013).

20. Hatchard, T. D. & Dahn, J. R. *In situ* XRD and electrochemical study of the reaction of lithium with amorphous silicon. *J. Electrochem. Soc.* **151,** A838–A842 (2004).

21. Ning, G., White, R. E. & Popov, B. N. A generalized cycle life model of rechargeable Li-ion batteries. *Electrochim. Acta* **51,** 2012–2022 (2006).

22. Umegaki, I. *et al.* Li-ion diffusion in Li intercalated graphite C_6Li and $C_{12}Li$ probed by μ^+SR. *Phys. Chem. Chem. Phys.* **19,** 19058–19066 (2017).

23. Strauß, F., Dörrer, L., Bruns, M. & Schmidt, H. Lithium tracer diffusion in amorphous Li_xSi for low Li concentrations. *J. Phys. Chem. C* **122,** 6508–6513 (2018).

24. Zhao, K. *et al.* Concurrent reaction and plasticity during initial lithiation of crystalline silicon in lithium-ion batteries. *J. Electrochem. Soc.* **159,** A238–A243 (2012).

25. Sethuraman, V. A., Chon, M. J., Shimshak, M., Srinivasan, V. & Guduru, P. R. *In situ* measurements of stress evolution in silicon thin films during electrochemical lithiation and delithiation. *J. Power Sources* **195,** 5062–5066 (2010).

26. Mao, Z., Farkhondeh, M., Pritzker, M., Fowler, M. & Chen, Z. Calendar aging and gas generation in commercial graphite/NCM-LMO lithium-ion pouch cell. *J. Electrochem. Soc.* **164,** A3469–A3483 (2017).

27. Huang, W. *et al.* Dynamic structure and chemistry of the silicon solid-electrolyte interphase visualized by cryogenic electron microscopy. *Matter* **1,** 1232–1245 (2019).

28. Louli, A. J., Ellis, L. D. & Dahn, J. R. Operando pressure measurements reveal solid electrolyte interphase growth to rank Li-ion cell performance. *Joule* **3,** 745–761 (2019).

29. Dose, W. M. *et al.* Capacity fade in high energy silicon-graphite electrodes for lithium-ion batteries. *Chem. Commun.* **54,** 3586–3589 (2018).

30. Su, X. *et al.* Silicon-based nanomaterials for lithium-ion batteries: a review. *Adv. Energy Mater.* **4,** 1300882 (2014).

31. Xu, L., Lu, C., Xu, Y. & Jia, J. Image smoothing via L_0 gradient minimization. *ACM Trans. Graphics* **30,** 174–184 (2011).

32. Serra, J. *Image Analysis and Mathematical Morphology*, vol. 1 (Academic Press, FL, 1983).

33. Otsu, N. A threshold selection method from gray-level histograms. *IEEE Trans. Cybern.* **9,** 62–66 (1979).

34. Abe, K., Colera, M., Shimamoto, K., Kondo, M. & Miyoshi, K. Functional electrolytes: recent advances in development of additives for resistance reduction. *J. Electrochem. Soc.* **161,** A863–A870 (2014).

35. Teufl, T., Pritzl, D., Solchenbach, S., Gasteiger, H. A. & Mendez, M. A. State of charge dependent resistance build-up in Li- and Mn-rich layered oxides during lithium extraction and insertion. *J. Electrochem. Soc.* **166,** A1275–A1284 (2019).

36. Obrovac, M. N. & Chevrier, V. L. Alloy negative electrodes for Li-ion batteries. *Chem. Rev.* **114,** 11444–11502 (2014).

37. Ko, M. *et al*. Scalable synthesis of silicon-nanolayer-embedded graphite for high-energy lithium-ion batteries. *Nat. Energy* **1,** 16113 (2016).

38. Gu, M. *et al*. *In situ* TEM study of lithiation behavior of silicon nanoparticles attached to and embedded in a carbon matrix. *ACS Nano* **6,** 8439–8447 (2012).

39. Liu, X. H. & Huang, J. Y. *In situ* TEM electrochemistry of anode materials in lithium ion batteries. *Energy Environ. Sci.* **4,** 3844–3860 (2011).

40. Yuan, Y., Amine, K., Lu, J. & Shahbazian-Yassar, R. Understanding materials challenges for rechargeable ion batteries with *in situ* transmission electron microscopy. *Nat. Commun.* **8,** 15806 (2017).

41. Sacci, R. *et al.* Nanoscale imaging of fundamental Li battery chemistry: solid-electrolyte interphase formation and preferential growth of lithium metal nanoclusters. *Nano Lett.* **15,** 2011–2018 (2015).

42. Holtz, M. E. *et al.* Nanoscale imaging of lithium ion distribution during in situ operation of battery electrode and electrolyte. *Nano Lett.* **14,** 1453–1459 (2014).

43. Zeng, Z. *et al.* Visualization of electrode–electrolyte interfaces in $LiPF_6$/EC/DEC electrolyte for lithium ion batteries via in situ TEM. *Nano Lett.* **14,** 1745–1750 (2014).

44. Gu, M. *et al.* Demonstration of an electrochemical liquid cell for operando transmission electron microscopy observation of the lithiation/delithiation behavior of Si nanowire battery anodes. *Nano Lett.* **13,** 6106–6112 (2013).

45. White, E. R., Lodico, J. J. & Regan, B. C. Intercalation events visualized in single microcrystals of graphite. *Nat. Commun.* **8,** 1969 (2017).

46. Langford, R. M. & Petford-Long, A. K. Preparation of transmission electron microscopy cross-section specimens using focussed ion beam milling. *J. Vac. Sci. Technol. A* **19,** 2186–2193 (2001).

47. Yuk, J. M., Seo, H. K., Choi, J. W. & Lee, J. Y. Anisotropic lithiation onset in silicon nanoparticle anode revealed by *in situ* graphene liquid cell electron microscopy. *ACS Nano* **8,** 7478–7485 (2014).

48. Cheong, J. Y. *et al.* Growth dynamics of solid electrolyte interphase layer on SnO_2 nanotubes realized by graphene liquid cell electron microscopy. *Nano Energy* **25,** 154–160 (2016).

49. Seo, H. K. *et al.* Strong stress-composition coupling in lithium alloy nanoparticles. *Nat. Commun.* **10,** 3428 (2019).

50. Liu, B. *et al.* Electron-beam radiation induced degradation of silicon nitride and its impact to semiconductor failure analysis by TEM. *AIP Adv.* **8,** 115327 (2018).
